# Estradiol Stimulates Vasodilatory and Metabolic Pathways in Cultured Human Endothelial Cells

**DOI:** 10.1371/journal.pone.0008242

**Published:** 2009-12-14

**Authors:** Agua Sobrino, Manuel Mata, Andrés Laguna-Fernandez, Susana Novella, Pilar J. Oviedo, Miguel Angel García-Pérez, Juan J. Tarín, Antonio Cano, Carlos Hermenegildo

**Affiliations:** 1 Research Foundation, Hospital Clínico Universitario, University of Valencia, Valencia, Spain; 2 Research Foundation, Hospital General Universitario, University of Valencia, Valencia, Spain; 3 Department of Physiology, University of Valencia, Valencia, Spain; 4 Department of Functional Biology and Physical Anthropology, University of Valencia, Valencia, Spain; 5 Department of Pediatrics, Obstetrics and Gynecology, University of Valencia, Valencia, Spain; Katholieke Universiteit Leuven, Belgium

## Abstract

Vascular effects of estradiol are being investigated because there are controversies among clinical and experimental studies. DNA microarrays were used to investigate global gene expression patterns in cultured human umbilical vein endothelial cells (HUVEC) exposed to 1 nmol/L estradiol for 24 hours. When compared to control, 187 genes were identified as differentially expressed with 1.9-fold change threshold. Supervised principal component analysis and hierarchical cluster analysis revealed the differences between control and estradiol-treated samples. Physiological concentrations of estradiol are sufficient to elicit significant changes in HUVEC gene expression. Notch signaling, actin cytoskeleton signaling, pentose phosphate pathway, axonal guidance signaling and integrin signaling were the top-five canonical pathways significantly regulated by estrogen. A total of 26 regulatory networks were identified as estrogen responsive. Microarray data were confirmed by quantitative RT-PCR in cardiovascular meaning genes; cyclooxigenase (COX)1, dimethylarginine dimethylaminohydrolase (DDAH)2, phospholipase A2 group IV (PLA2G4) B, and 7-dehydrocholesterol reductase were up-regulated by estradiol in a dose-dependent and estrogen receptor-dependent way, whereas COX2, DDAH1 and PLA2G4A remained unaltered. Moreover, estradiol-induced COX1 gene expression resulted in increased COX1 protein content and enhanced prostacyclin production. DDAH2 protein content was also increased, which in turn decreased asymmetric dimethylarginine concentration and increased NO release. All stimulated effects of estradiol on gene and protein expression were estrogen receptor-dependent, since were abolished in the presence of the estrogen receptor antagonist ICI 182780. This study identifies new vascular mechanisms of action by which estradiol may contribute to a wide range of biological processes.

## Introduction

The incidence of coronary heart disease is greater in men than in premenopausal women of the same age, but increases in frequency after menopause, an effect that has been attributed, at least in part, to estrogens [1]. Estrogens have been used as contraceptive agents or as principal constituents of hormone replacement therapy formulations in postmenopausal women, 17β-estradiol being the most widely used molecule. The cardiovascular protective effect detected in a considerable number of observational clinical studies [2] has not been confirmed by more recent randomized placebo-controlled trials designed to study the effects of hormonal therapy in either secondary [3], [4] or primary [5] prevention. It should be stated that the clinical trials of estrogen therapy for the treatment of cardiovascular disease are largely flawed (e.g., hormone replacement therapy started too late in menopause). Moreover, a number of studies have demonstrated a favorable profile for estrogens in both experimental animal as well as *in vitro* models [6].

Endothelium is crucial to the modulation of vessel tone and to the control of platelet adhesion and aggregation, two key factors in the initiation and development of atherosclerosis [7]. Endothelium, including human umbilical vein endothelial cells (HUVEC), expresses both types of estrogen receptors (ER), α and β, and the actions of estrogens on endothelium have been exhaustively studied [8]. Moreover, clinical and experimental data support the consideration of endothelium as a target for sexual hormones [9]. Estradiol effects on partial gene expression in endothelial cells have frequently been studied, but there is a lack about its effects on the whole gene expression profile.

Microarrays are high-throughput genomic tools that allow the comparison of global expression changes in thousands of genes between different experimental conditions in cell/tissue analysis, and they have been widely adopted for analyzing the global gene expression profiles *in vivo* and *in vitro* [10]. Recent studies have demonstrated the ability of this technology for investigating molecular pathophysiological mechanisms involved in a variety of human diseases. For instance, microarray technology has been used as a novel experimental approach to analyze alterations in gene expression in different cardiovascular diseases [11], atherosclerosis [12] and experimental stroke in rats [13].

Microarray technology offers the possibility of exploring a large number of candidate genes which are modified by estrogens. The present study aims to explore gene expression modification, mainly focused on candidate genes that may regulate the vascular effects of estrogens, in cultured human endothelial cells exposed to physiological concentrations of estradiol by using microarrays, thus providing new information to the available body of knowledge about the influence of estradiol on the vascular wall.

## Materials and Methods

### Ethics Statement

This investigation conforms to the principles outlined in the Declaration of Helsinki, was approved by the Ethical Committee of Clinical Research of the University Clinical Hospital of Valencia, and written informed consent was obtained from all donors.

### Cell Culture and Experimental Design

Primary HUVEC were isolated, grown, and identified as described earlier [14] in human endothelial cell-specific Medium EBM-2 (Lonza, Basel, Switzerland), supplemented with EGM-2 (Lonza), in an incubator at 37°C with 5% CO_2_.

Cells from passages 4 to 6 were seeded onto 25 cm^2^ flasks for mRNA isolation. When cells were at 75% of confluence, culture medium was exchanged for a phenol red–free Medium 199 (GIBCO, Invitrogen, Barcelona, Spain) supplemented with 20% charcoal/dextran-treated fetal bovine serum (GIBCO), EGM-2, pyruvic acid and antibiotics (“hormone free medium”) to avoid any estrogenic activity and maintained for 24 hours. Then, culture medium was eliminated and replaced by phenol red-free medium 199.

Cells were exposed to different concentrations (range: 0,01 – 100 nmol/L) of 17β-estradiol (Sigma, Alcobendas, Spain) by serial dilutions of a stock solution with phenol red-free medium. The pure anti-estrogen ICI182780 (1 µmol/L; Biogen, Madrid, Spain) was used to evaluate whether the observed effects were mediated by ER modulation. Control cells were exposed to the same vehicles of estradiol (0.1% ethanol) or ICI182780 (0.1% DMSO). All treatments were added in hormone free medium and experiments were performed at 75–80 % of confluence.

### RNA Isolation and Genechip Expression Analysis

To carry out the microarray experiments, HUVEC from 9 separate cultures were exposed to control (0.1% ethanol) and 1 nmol/L estradiol treatments for 24 h. Total cellular RNA was extracted by using the TRIzol® reagent (Invitrogen, USA) following the manufacturer's instructions. RNA integrity was assayed by means of the 2100 Bioanalizer (Agilent Technologies, Santa Clara, CA, USA). Equal amounts of RNA extracted from 3 control- or 3 estradiol-treated cultured flasks obtained from three different cultures were pooled, achieving three biological replicates of the control and three that were treated with estradiol. Therefore, a total number of 6 microarrays were developed (3 control pools, named C1, C2, C3, and 3 estradiol-treated pools named E1, E2 and E3). Five micrograms of total RNA were amplified and labeled according to GeneChip Expression Analysis Technical Manual (Affymetrix Ltd, UK). The concentration of biotinylated and fragmented cRNA was measured using the 2100 Bioanalizer (Agilent Technologies). Twenty micrograms of fragmented biotinylated cRNA were used to prepare the hybridization cocktail and subsequently hybridized for sixteen hours to the Human Genome U133A plus 2.0 microarrays, which analyzes the expression level of over 47000 transcripts and variants. Arrays were washed and stained according to the EukGene_ws_2v5 in the Fluidics Station (Affymetrix) and scanned using the GeneChip scanner 3000. Affymetrix's GeneChip Operating Software (GCOS, Affymetrix) was used to obtain and analyze images.

Files obtained from GCOS (.cel) were used to analyze significant changes in expression profiles of different experimental groups using the dCHIP Analysis Software and the SpotFire Decision Site software. Data were normalized using the Invariant Set Method described earlier [15] and modeled using the PM/MM model. Then, ANOVA was used to find significant changes among experimental groups. False Discovery Rate (FDR) was used to discriminate false positives in the multivariant system. Only adjusted p-values <0.05 were considered significant. Global differences between different samples were measured by Principal Component Analysis (PCA) and Linear Discrimination Analysis (LDA). Hierarchical Cluster was used to analyze expression profiles of different samples, and was carried out using UPGMA (Unweighted Pair Group Method with Arithmetic Mean) analysis, with an ordering function based on the input rank. Data are represented as a dendrogram, with the closest branches of the tree representing arrays with similar gene expression patterns. Gene Onthology Browser (Nettaffyx Analysis Center, Affymetrix) was used to classify genes according to functionality context. Finally, relationships among data were screened using the Pathway Architect software (Stratagene, La Joya, CA, USA). All data discussed in this publication is MIAME compliant and that the raw data has been deposited in NCBI's Gene Expression Omnibus [16], a MIAME compliant database, and are accessible through GEO Series accession number GSE16683.

### Network Identification and Canonical Pathway Analysis

List of genes significantly regulated by estrogen were analyzed using Ingenuity Pathways Analysis (IPA) software (Ingenuity Systems, Redwood City, CA, USA). IPA uses a variety of computational algorithms to identify and establish cellular networks that statistically fit the input gene list and expression values from experiments. Data sets containing the Affymetrix probe set identifiers and fold changes of genes were overlaid onto a global molecular network developed from information contained in the database. Networks were then algorithmically generated based on their connectivity and a score was assigned. The score is used to rank networks according to how relevant they are to the genes in the input dataset. Each network or pathway was arbitrarily set to have a maximum of 35 focus genes. Genes or gene products are represented as nodes, and the biological relationship between two nodes is represented as an edge. The intensity of the node color indicates the degree of up- (red) or down- (green) regulation.

Canonical pathways analysis identified the pathways, which were most significant to the input data set. The significance of the association between the data set and the canonical pathway was determined based on two parameters: (1) a ratio of the number of genes from the data set that map to the pathway divided by the total number of genes that map to the canonical pathway and (2) a P value calculated using Fischer's exact test determining the probability that the association between the genes in the data set and the canonical pathway is due to chance alone.

### Quantitative Real Time PCR (QRT-PCR) Assays

Reverse transcription (RT) was carried out using SuperScript^TM^ II Synthesis System for RT–PCR (Invitrogen) by using a personal Mastercycler Eppendorf Thermocycler (Eppendorf, Hamburg, Germany). Different samples than that used for microarrays experiments were used to perform the QRT-PCR assays. One microgram of total RNA was reverse-transcribed to cDNA following the manufacturer's instructions. For each RT, a blank was prepared using all the reagents except the RNA sample (replaced with an equivalent volume of diethylpyrocarbonate (DEPC)-treated water) and also used as non-template control in real-time PCR experiments.

Quantitative real-time PCR (QRT-PCR) was done with SYBR-Green PCR Master Mix or TaqMan Universal Mastermix (Applied Biosystems, Fosters City, CA, USA). In the case of DHCRA7, PLA2G4A and PLA2G4B, the PCR reaction mix was prepared in 0.2 mL RNase free tubes by adding a volume of TaqMan Universal PCR Master Mix and TaqMan Gene Expression Assay (Table 1). The sample of cDNA obtained from the RT was incorporated into the necessary quantity of DEPC-treated water to get a final concentration of 40 ng approximately (range: 10–100 ng). The GADPH gene was used as endogenous control. The appropriate volume of each reaction mixture was transferred to a reaction plate which was then placed in the 7900HT Fast Real-Time PCR System (Applied Biosystems) with the appropriate thermal cycling conditions (50°C/2 min, 95°C/10 min, 40 Cycles; 95°C/15 s, 6°C/1 min).

**Table 1:** List of abbreviations and primers used for RT-PCR.

Gene	Abbreviation	Accession no.	Custom Primer	Sequence	Fragment (bp)
Ciclooxigenase-1	COX-1	AF440204	Forward Reverse	5′-TACTCACAGTGCGCTCCAAC-3′ 5′-GCAACTGCTTCTTCCCTTTG-3′	168
Ciclooxigenase-2	COX-2	D28235	Forward Reverse	5′-ATCATAAGCAGGGCCAGCT-3′ 5′-AAGGCGCAGTTTACGCTGTC-3′	101
Dimethylarginine dimethylaminohydrolase-1	DDAH-1	BC_033680	Forward Reverse	5′-GGACAAATCAACGAGGTGCT-3′ 5′-TAGCGGTGGTCACTCATCTG-3′	193
Dimethylarginine dimethylaminohydrolase-2	DDAH-2	NM_013974	Forward Reverse	5′-GATCTGGCCAAAGCTCAAAG-3,5′-CAACCCAGGACGAAGAAAGA-3′	573
*Glyceraldehyde 3-phosphate dehydrogenase*	*GADPH*	NM_002046	Forward Reverse	5′-CTGCTCCTCCTGTTCGACAGT-3′ 5′-CCGTTGACTCCGACCTTCAC-3′	100

Gene	Gene	Accesion no.	TaqMan® Gene Expression Assays	Fragment (bp)
7-dehydrocholesterol reductase	DHCR7	NM_001360.2	Hs00154732_m1	85
Phospholipase A2, group IVA	PLA2G4A	NM_024420.2	Hs00233352_m1	69
Phospholipase A2, group IVB	PLA2G4B	NM_005090.2	Hs00192661_m1	71
*Glyceraldehyde 3-phosphate dehydrogenase*	*GADPH*	NM_002046.3	endogenous control	122

In the case of COX-1, COX-2, DDAH-1 and DDAH-2, a QRT-PCR was performed using an ABI PRISM 7700 Sequence Detection System (Applied Biosystems) with a heated lid (105°C), an initial denaturation step at 95°C for 10 min, followed by 40 cycles of 95°C for 15 s and 60°C for 1 min. To amplify cDNA, the RT samples were diluted 1/10. In each reaction, a total of 1 µL from each RT tube was mixed with 12.5 µL of SYBR Green PCR master mix (Applied Biosystems) containing nucleotides, Taq DNA polymerase, MgCl_2_ and reaction buffer with SYBR green; 1.5 µL of 5 µmol/L adequate primers and DEPC-treated water were added to a final volume of 25 µL. In parallel, 5-fold serial dilutions of well-known DNA concentrations were run as calibration curves. Primers (Table 1) were designed using the Primers Express Software (Applied Biosystems) and synthesized by Custom Primers (Life Technologies, Barcelona, Spain).

Data were analysed with the ABI PRISM Sequence Detection v. 1.7 analysis software (Perkin Elmer, Nieuwerkerk, The Netherlands). To validate a QRT-PCR, standard curves with r>0.95 and slope values between −3.1 and −3.4 were required. Gene expression was relative quantified based on the work of Pfaffl [17]. In some samples, PCR bands were purified using a MiniElute PCR Purification Kit (Qiagen, Valencia, CA, USA) and then sequenced to prove that the amplified products corresponded to previously published sequences. Agarose gel electrophoreses were also performed to demonstrate that QRT-PCR yielded a unique band.

### Immunoblotting

HUVEC were treated in 25 cm^2^ flasks for 24 hours with the desired products. A volume of 150 µL of lysis buffer (0.1 % triton X-100, 0.5 % sodium deoxicholate acid, 0.1 % Sodium Dodecyl Sulphate (SDS), 0.1% phenylmethanesulphonylfluoride or phenylmethylsulphonyl fluoride (PMSF), in 100 mL of phosphate saline buffer (PBS) containing protease inhibitors: 1 µg/mL leupeptin, 0.5 µg/mL pepstatin and 1 µg/mL bestatin) was added and maintained at 4°C for 30 minutes. Then, cells were collected using a cell scraper, boiled for 5 minutes and sonicated for 10 seconds. Protein content was measured [18] and samples were frozen at –20°C until assay.

Equal amounts of protein (60–80 µg) were then separated by 10% of SDS-Polyacrylamide gel electrophoresis, and the protein was transferred to PVDF sheets (Biorad, Spain). Immunostaining was achieved using specific antibodies anti-ERα (sc-8002; Santa Cruz Biotechnology, Santa Cruz, CA, USA), anti-ERβ (sc-8974; Santa Cruz Biotechnology), anti-COX-1 (cat 236003; Calbiochem, Germany), anti-COX-2 (cat 160107; Cayman Chemical), anti-DDAH-I (PC716; Calbiochem) or anti-DDAH-II (PC717; Calbiochem). Development was performed with alkaline-phosphatase-linked appropriate secondary antibodies (from Sigma), followed with nitroblue tetrazolium (NBT)/5-Bromo-4-Chloro-3-Indolyl Phosphate, p-toluidine salt (BCIP) color development reaction. Blots were digitalized using a Gelprinter PLUS (TDI, Madrid, Spain), and the densities of spots were analyzed with the program Image Gauge 4.0 (Science Lab. 2001). Equivalent protein loading and transfer efficiency were verified by staining for β-actin (Sigma).

### Prostacyclin Assay

After treatment with the desired products, medium was collected and stored at –20°C until prostacyclin was measured. Culture wells were then washed with PBS and adherent cells were collected in 0.5 N NaOH for protein determination by the modified Lowry's method using bovine serum albumin as standard [18].

The amount of prostacyclin produced, calculated as the concentration of stable hydrolysis product, 6-keto-prostaglandin-F1α, was assessed in duplicate by a commercial EIA kit (Cayman Chemical). Prostacyclin production was expressed as ng prostacyclin/mg protein.

### Isolation and Measurement of Asymmetric Dimethylarginine (ADMA)

After 24 hours of incubation with the desired treatments, medium was collected and stored at –20°C until asymmetric dimethylarginine (ADMA), a major endogenous inhibitor of nitric oxide synthase (NOS), quantification. Culture wells were then washed with PBS and adherent cells were collected in 0.5 N NaOH solution for protein determination [18].

Measurement of ADMA was accomplished by high-performance liquid chromatography (HPLC) as described earlier [19]. In brief, ADMA from 1 mL of culture medium was purified with Bond Elut SCX columns (Varian Inc., Palo Alto, CA, USA) and eluted with 4 mL of methanol containing 30% distilled water and 2% triethylamine. The eluent was then evaporated to dryness at 60°C, and the dried extract was redissolved in running buffer. HPLC was carried out on a Shimadzu chromatography system (Shimadzu Corporation, Kyoto, Japan). Separation of ADMA was achieved with a 250×4.6-mm (inner diameter), 5-µm “Kromasil C18” analytical column (Scharlau, Barcelona, Spain) using 25 mM phosphoric acid containing 10 mM hexane sulphonic acid and 1 % [v/v] acetonitrile, pH 5.0. The analysis was carried out at a flow rate of 1.3 mL/min and the absorbance monitored at 200 nm. Concentrations of ADMA in the samples were determined by comparison with standards (Sigma, Alcobendas, Spain). ADMA production was expressed as nmol/mg protein.

### Nitric Oxide (NO) Production

After 24 hours of incubation with the desired treatments, cells were washed and incubated with HEPES buffer (5 mM HEPES containing (in mM) 140 NaCl, 5 KCl, 2 CaCl_2_, 1 MgCl_2_ and 10 glucose, pH adjusted to 7.4), for 120 min. Then, incubation medium was collected, culture wells were washed with PBS, and adherent cells were collected in 0.5 N NaOH solution for protein determination [18].

Endothelial NO production was determined in culture medium using the ISONOP nitric oxide sensor (World Precision Instruments, Sarasota, FL, USA), an amperometric sensor specific for NO, as described earlier [19]. A chemical titration calibration was performed with use of an acidic iodide solution (0.1 mol/l H_2_SO_4_, 0.14 mol/l K_2_SO_4_, 0.1 mol/l KI) against varied volumes of KNO_2_. NO was formed stoichiometrically and measured directly. The quantity of NO was expressed as nmol/mg protein.

### Statistical Analysis

Values shown in the text and figures are mean ± SEM. ANOVA test was applied for comparisons of mean, and then Bonferroni's test was performed. P values<0.05 were considered significant. The statistical analysis was carried out using the Prism 4 software (GraphPad Software Inc., San Diego, CA, USA).

## Results

### Identification of Global Gene Expression Changes in Estradiol–Treated HUVEC

The gene expression profile of human vascular cells treated with or without estradiol was assessed by using the Human Genome U133A plus 2.0 microarray technology from Affymetrix. A total of 1886 genes passed the ANOVA analysis, with fold changes between 2.99 and −5.34. Table 2 (online supporting information) summarizes most differentially expressed genes between control and estradiol treated samples. Only genes with more than a 1.9-fold change were included. As expected, the list of genes became greater as a more permissive fold-change was selected. Only 4 genes (∼14%) were up-regulated and 25 (∼86%) were down-regulated when the fold-change was higher than 2.5. By decreasing the fold-change, the number of genes regulated by estradiol increased, and there was a tendency to equate the percentage of genes up-regulated and down-regulated. For instance, with a fold-change higher than 1.9, 187 genes were significantly regulated: 95 (∼50%) were up-regulated and 92 (∼50%) were down-regulated.

**Table 2:** Genes that changed more than 1.9-fold with estradiol.

probe set	gene	Accession	Control mean	Control SD	Estradiol mean	Estradiol SD	fold change	P value
212969_x_at	hypothetical protein FLJ35827	BE222618	175,19	19,45	500,79	31,72	2,86	0,00342
223967_at	angiopoietin-like 6	AF230330	30,98	8,72	86,64	5,75	2,8	0,02964
212064_x_at	MYC-associated zinc finger protein (purine-binding transcription factor)	AI471665	186,55	15,26	505,1	107,26	2,71	0,03497
203442_x_at	hypothetical protein FLJ35827	AA478965	195,81	7,93	504,02	44,58	2,57	0,01052
224182_x_at	sema domain, transmembrane domain (TM), and cytoplasmic domain, (semaphorin) 6B	AF293363	68,13	17,43	169,76	12,74	2,49	0,01574
209079_x_at	protocadherin gamma subfamily C, 3	AF152318	203,67	43,44	503,57	126,44	2,47	0,02558
227463_at	angiotensin I converting enzyme (peptidyl-dipeptidase A) 1	AW057540	69,93	12,41	170,95	17,15	2,44	0,03837
200707_at	protein kinase C substrate 80K-H	NM_002743	221,54	57,51	531,35	19,47	2,4	0,01438
240786_at	Notch homolog 4 (Drosophila)	AI341271	52,66	5,54	125	14,98	2,37	0,03691
204693_at	CDC42 effector protein (Rho GTPase binding) 1	NM_007061	115,37	10,39	270,64	34,17	2,35	0,02829
201050_at	phospholipase D3	NM_012268	274,9	34,99	647,38	19,89	2,35	0,00206
201396_s_at	small glutamine-rich tetratricopeptide repeat (TPR)-containing, alpha	NM_003021	166	16,76	389,34	54,20	2,35	0,01439
202017_at	epoxide hydrolase 1, microsomal (xenobiotic)	NM_000120	85,85	19,59	200,8	23,62	2,34	0,04111
227753_at	hypothetical protein FLJ90586	R26843	42,02	6,69	98,19	3,76	2,34	0,01451
230698_at	MRNA; cDNA DKFZp434H205 (from clone DKFZp434H205)	AW072102	42,87	8,54	100,33	23,38	2,34	0,04531
208611_s_at	spectrin, alpha, non-erythrocytic 1 (alpha-fodrin)	U83867	296,27	32,51	689,31	134,18	2,33	0,04892
209235_at	chloride channel 7	AL031600	93,14	14,31	214,55	28,67	2,3	0,02221
211136_s_at	cleft lip and palate associated transmembrane protein 1	BC004865	97,85	34,88	223,39	2,62	2,28	0,03365
209427_at	smoothelin	AF064238	148,48	18,65	337,87	36,31	2,28	0,01198
200859_x_at	filamin A, alpha (actin binding protein 280)	NM_001456	837,96	143,55	1894,68	238,65	2,26	0,00676
224792_at	tankyrase 1 binding protein 1, 182 kDa	AL566438	83,59	21,44	188,84	16,91	2,26	0,03592
212127_at	Ran GTPase activating protein 1	BE379408	148,57	70,00	322,79	15,80	2,17	0,04023
230112_at	membrane-associated ring finger (C3HC4) 4	AB037820	181,14	83,07	391,8	35,54	2,16	0,02505
1570318_at	Homo sapiens, clone IMAGE:4792986, mRNA	BC030089	69,31	35,11	148,72	19,06	2,15	0,01849
205185_at	serine protease inhibitor, Kazal type 5	NM_006846	160,85	55,95	346,46	19,02	2,15	0,01393
222206_s_at	nicalin homolog (zebrafish)	AA781143	91,29	8,04	195,18	9,50	2,14	0,0149
201373_at	plectin 1, intermediate filament binding protein 500 kDa	NM_000445	177,33	23,86	379,57	15,39	2,14	0,01277
1552667_a_at	SH2 domain containing 3C	NM_005489	95,12	13,48	203,46	16,70	2,14	0,04872
209051_s_at	ral guanine nucleotide dissociation stimulator	AF295773	96,35	14,24	205,2	3,85	2,13	0,02245
211564_s_at	PDZ and LIM domain 4	BC003096	171,62	16,57	364,69	6,42	2,12	0,00636
240350_at	Transcribed locus	AI769817	70,76	23,81	148,99	13,00	2,11	0,0374
201797_s_at	valyl-tRNA synthetase 2	NM_006295	95,72	15,28	201,89	17,35	2,11	0,03045
210428_s_at	hepatocyte growth factor-regulated tyrosine kinase substrate	AF260566	258,31	9,63	543,12	10,09	2,1	0,00539
202855_s_at	solute carrier family 16 (monocarboxylic acid transporters), member 3	AL513917	63,41	16,15	132,88	2,87	2,1	0,03614
202320_at	general transcription factor IIIC, polypeptide 1, alpha 220 kDa	NM_001520	90,81	7,28	189,55	4,47	2,09	0,00867
218051_s_at	hypothetical protein FLJ12442	NM_022908	321,65	53,09	667,99	45,65	2,08	0,00553
200808_s_at	zyxin	NM_003461	423,14	13,27	875,16	138,22	2,07	0,02725
216267_s_at	placental protein 6	BF034906	114,34	23,47	234,61	1,02	2,05	0,02765
219270_at	hypothetical protein MGC4504	NM_024111	128,82	44,89	262,36	58,64	2,04	0,04536
219922_s_at	latent transforming growth factor beta binding protein 3	NM_021070	323,66	30,45	661,67	37,35	2,04	0,01075
1564494_s_at	procollagen-proline, 2-oxoglutarate 4-dioxygenase (proline 4-hydroxylase), beta polypeptide	AK075503	174,37	52,22	355,76	10,91	2,04	0,04303
201251_at	pyruvate kinase, muscle	NM_002654	417,25	18,67	851,66	107,61	2,04	0,02554
45714_at	host cell factor C1 regulator 1 (XPO1 dependant)	AA436930	136,61	17,78	276,98	20,76	2,03	0,02748
212359_s_at	KIAA0913	W89120	155,23	17,17	314,75	29,07	2,03	0,01367
201168_x_at	Rho GDP dissociation inhibitor (GDI) alpha	NM_004309	384,7	36,45	780,38	119,07	2,03	0,023
223383_at	zinc and ring finger 1	AL136903	95,87	25,43	195,09	6,64	2,03	0,04785
215909_x_at	misshapen-like kinase 1 (zebrafish)	AL157418	145,34	48,17	294,11	68,43	2,02	0,01318
35265_at	fragile X mental retardation, autosomal homolog 2	AF044263	100,19	6,16	201,48	3,24	2,01	0,01011
226367_at	Jumonji, AT rich interactive domain 1A (RBBP2-like)	AA854032	57,11	11,39	114,98	15,75	2,01	0,04725
229192_s_at	tubulin-specific chaperone d	AL096745	170,61	43,32	342,84	9,29	2,01	0,02167
218522_s_at	BPY2 interacting protein 1	NM_018174	165,12	25,92	330,46	5,94	2	0,01544
200766_at	cathepsin D (lysosomal aspartyl protease)	NM_001909	113,56	1,19	227,52	15,59	2	0,02324
222155_s_at	G protein-coupled receptor 172A	AK021918	74,97	10,34	149,89	2,16	2	0,01724
227347_x_at	hairy and enhancer of split 4 (Drosophila)	NM_021170	79,62	26,99	159,32	25,99	2	0,03086
215807_s_at	plexin B1	AV693216	72,17	16,35	144,65	1,99	2	0,03366
202161_at	protein kinase N1	NM_002741	236,28	21,45	472,79	10,07	2	0,0105
217937_s_at	histone deacetylase 7A	NM_016596	215,95	32,53	429,04	8,40	1,99	0,01042
209166_s_at	mannosidase, alpha, class 2B, member 1	U68567	199,46	45,12	396,59	46,73	1,99	0,01271
212968_at	radical fringe homolog (Drosophila)	BF940276	159,88	10,60	317,92	31,31	1,99	0,01304
209651_at	transforming growth factor beta 1 induced transcript 1	BC001830	934,12	83,13	1863,42	337,88	1,99	0,02541
203926_x_at	ATP synthase, H+ transporting, mitochondrial F1 complex, delta subunit	NM_001687	330,84	24,63	655,04	26,25	1,98	0,00858
208890_s_at	plexin B2	BC004542	258,45	52,59	510,68	26,53	1,98	0,0126
217007_s_at	a disintegrin and metalloproteinase domain 15 (metargidin)	AK000667	101,77	21,65	200,18	6,12	1,97	0,02402
201360_at	cystatin C (amyloid angiopathy and cerebral hemorrhage)	NM_000099	384,94	8,11	758,71	49,24	1,97	0,01031
223050_s_at	F-box and WD-40 domain protein 5	BC000850	107,78	10,49	212,4	22,07	1,97	0,02375
208132_x_at	HLA-B associated transcript 2	NM_004638	117,46	6,61	230,97	11,34	1,97	0,01343
201264_at	coatomer protein complex, subunit epsilon	NM_007263	221,17	57,65	434,02	18,52	1,96	0,04857
210622_x_at	cyclin-dependent kinase (CDC2-like) 10	AF153430	56,4	9,55	110,79	6,37	1,96	0,04874
209729_at	growth arrest-specific 2 like 1	BC001782	133,53	13,55	261,57	28,58	1,96	0,02885
201102_s_at	phosphofructokinase, liver	NM_002626	201,25	8,94	394,24	6,59	1,96	0,0074
201281_at	adhesion regulating molecule 1	NM_007002	349,38	56,97	679,65	108,43	1,95	0,02532
221009_s_at	angiopoietin-like 4	NM_016109	224,97	19,81	438,78	85,29	1,95	0,03765
214175_x_at	PDZ and LIM domain 4	AI254547	264,5	24,73	514,92	27,01	1,95	0,00287
203055_s_at	Rho guanine nucleotide exchange factor (GEF) 1	NM_004706	136,27	20,25	265,87	27,67	1,95	0,03729
201079_at	synaptogyrin 2	NM_004710	423,03	23,29	826,04	79,77	1,95	0,01402
244017_at	Tax1 (human T-cell leukemia virus type I) binding protein 1	AI218142	73,03	13,78	142,55	9,52	1,95	0,04703
40829_at	WD and tetratricopeptide repeats 1	AB028960	114,71	11,89	223,14	33,03	1,95	0,0312
201945_at	furin (paired basic amino acid cleaving enzyme)	NM_002569	186,14	26,50	361,57	25,35	1,94	0,04325
222003_s_at	dedicator of cytokinesis 6	BE857715	141,94	34,44	273,32	3,82	1,93	0,02743
200747_s_at	nuclear mitotic apparatus protein 1	NM_006185	177,05	30,13	342,53	1,12	1,93	0,02273
218494_s_at	SLC2A4 regulator	NM_020062	164,72	22,13	317,53	22,57	1,93	0,01816
217729_s_at	amino-terminal enhancer of split	NM_001130	200,99	28,32	386,1	14,93	1,92	0,02748
208978_at	cysteine-rich protein 2	U36190	522,29	187,11	1003,95	51,34	1,92	0,02812
204355_at	DEAH (Asp-Glu-Ala-His) box polypeptide 30	NM_014966	216,71	19,48	415,91	36,10	1,92	0,0208
226307_at	transducer of regulated cAMP response element-binding protein (CREB) 2	AW504757	183,05	15,21	351,43	67,63	1,92	0,03444
204431_at	transducin-like enhancer of split 2 (E(sp1) homolog, Drosophila)	NM_003260	191,78	47,68	368,22	11,00	1,92	0,03922
223179_at	yippee-like 3 (Drosophila)	BC005009	131,63	33,05	253,07	4,77	1,92	0,037
214679_x_at	guanine nucleotide binding protein (G protein), alpha 11 (Gq class)	AL110227	191,38	15,11	365,57	52,83	1,91	0,0273
205740_s_at	hypothetical protein MGC10433	NM_024321	140,3	11,74	268,07	12,03	1,91	0,01253
208110_x_at	mediator of RNA polymerase II transcription, subunit 25 homolog (yeast)	NM_030973	120,74	11,71	231,04	22,23	1,91	0,01676
227557_at	scavenger receptor class F, member 2	AI127800	69,68	7,47	133,39	3,34	1,91	0,01744
203254_s_at	talin 1	NM_006289	302,17	24,41	576,14	97,40	1,91	0,02533
225868_at	tripartite motif-containing 47	AW249467	260,23	15,67	497,05	68,76	1,91	0,03083
217912_at	PP3111 protein	NM_022156	301,95	39,31	573,85	26,94	1,9	0,01004
219802_at	hypothetical protein FLJ22028	NM_024854	247,99	16,16	129,67	17,21	−1,91	0,02723
220553_s_at	PRP39 pre-mRNA processing factor 39 homolog (yeast)	NM_018333	348,68	49,92	182,71	2,12	−1,91	0,03766
222129_at	Chromosome 2 open reading frame 17	AK026155	475,98	66,26	248,54	70,12	−1,92	0,00982
209525_at	Hepatoma-derived growth factor, related protein 3	BG285017	399,99	13,42	208,81	72,27	−1,92	0,04455
214101_s_at	Aminopeptidase puromycin sensitive	BG153399	518,57	14,39	267,18	60,68	−1,94	0,0284
201694_s_at	early growth response 1	NM_001964	912,16	116,79	467,6	123,62	−1,95	0,00691
1559391_s_at	UDP-Gal:betaGlcNAc beta 1,4- galactosyltransferase, polypeptide 5	AI084451	301,03	26,43	154,27	23,44	−1,95	0,03508
228193_s_at	Response gene to complement 32	AI744499	250,61	22,33	127,67	56,97	−1,96	0,04859
1556019_at	hypothetical protein LOC144874	BE502765	1173,2	95,58	596,16	173,35	−1,97	0,01607
230206_at	Dedicator of cytokinesis 5	AI692645	158,44	36,03	79,79	11,89	−1,99	0,0427
229397_s_at	Glucocorticoid receptor DNA binding factor 1	AI275597	147,21	12,14	74,15	11,65	−1,99	0,04387
1555272_at	hypothetical protein LOC285927	BC044242	618,29	51,90	310,14	155,33	−1,99	0,04207
229420_at	Similar to 60S ribosomal protein L23a	AI557425	1107,1	154,44	557,27	237,67	−1,99	0,02051
219375_at	choline	NM_006090	433,25	69,86	216,49	13,98	−2	0,02552
229787_s_at	O-linked N-acetylglucosamine (GlcNAc) transferase	AI742039	407,25	82,20	203,63	53,17	−2	0,01368
216246_at	Ribosomal protein S20	AF113008	718,07	204,97	359,53	80,17	−2	0,03995
1552664_at	folliculin	NM_144997	315,35	34,67	156,57	10,05	−2,01	0,01331
1558504_at	Similar to hypothetical protein LOC284701	AF086554	161,72	10,90	79,91	27,44	−2,02	0,0256
235918_x_at	gb:AL559474	AL559474	120,53	29,85	59,5	8,65	−2,03	0,0485
225920_at	hypothetical protein LOC148413	AW452640	735,04	27,11	362,24	119,58	−2,03	0,0301
229538_s_at	IQ motif containing GTPase activating protein 3	AW271106	161,6	20,81	79,43	18,07	−2,03	0,01163
221910_at	hypothetical protein LOC221810	BF131965	152,28	9,84	74,67	20,84	−2,04	0,03427
229630_s_at	Wilms tumor 1 associated protein	AU147416	1450,7	50,43	712,54	184,74	−2,04	0,01547
218737_at	sno, strawberry notch homolog 1 (Drosophila)	NM_018183	116,38	20,25	56,81	4,58	−2,05	0,04833
1561481_at	Homo sapiens, clone IMAGE:4827393, mRNA	BC034606	271,34	79,40	131,55	34,12	−2,06	0,04476
206448_at	zinc finger protein 365	NM_014951	198,83	24,74	96,55	1,99	−2,06	0,02286
238002_at	golgi phosphoprotein 4	BF342391	886,71	127,65	428,76	97,91	−2,07	0,00471
204863_s_at	interleukin 6 signal transducer (gp130, oncostatin M receptor)	BE856546	135,03	26,71	65,32	18,57	−2,07	0,03394
243159_x_at	Myosin X	AI247495	363,27	42,74	175,08	38,42	−2,07	0,00176
234344_at	RAP2C, member of RAS oncogene family	AF093744	267,15	21,81	128,37	30,05	−2,08	0,01344
214038_at	chemokine (C-C motif) ligand 8	AI984980	168,57	27,35	79,65	5,77	−2,12	0,02801
242467_at	Full-length cDNA clone CS0DJ012YP16 of T cells (Jurkat cell line)	BF433200	213,36	47,26	100,12	8,02	−2,13	0,04832
224346_at	gb:AF116671.1	AF116671	245,39	57,23	114,94	37,07	−2,13	0,01828
241872_at	Hypothetical protein DKFZp761D221	AI149963	354,96	59,53	165,78	12,12	−2,14	0,0268
202425_x_at	protein phosphatase 3 (formerly 2B), catalytic subunit, alpha isoform (calcineurin A alpha)	NM_000944	837,16	7,08	391,06	61,98	−2,14	0,0101
235999_at	Heterogeneous nuclear ribonucleoprotein D (AU-rich element RNA binding protein 1, 37 kDa)	AA863112	136,83	22,52	63,56	19,97	−2,15	0,02282
217550_at	Activating transcription factor 6	AA576497	517,27	117,65	238,89	64,33	−2,17	0,0154
1565765_x_at	Hypothetical protein FLJ14834	AL832478	618,76	41,26	285,03	110,71	−2,17	0,02286
219138_at	ribosomal protein L14	BC000606	568,98	102,87	262,25	146,81	−2,17	0,02015
228545_at	Zinc finger protein 148 (pHZ-52)	AI016784	166,27	30,20	76,32	7,03	−2,18	0,04509
239376_at	CDNA clone IMAGE:4333081, partial cds	AA489041	244,11	20,12	111,45	30,03	−2,19	0,04759
201159_s_at	N-myristoyltransferase 1	NM_021079	98,32	10,08	44,9	7,35	−2,19	0,04858
240145_at	Transcribed locus, moderately similar to NP_008471.1 Canis familiaris ND1 gene	AW628059	118,98	22,05	54,36	14,20	−2,19	0,04816
238608_at	Laminin, beta 1	AI174988	301,52	55,25	136,99	40,66	−2,2	0,00821
244356_at	Protein tyrosine phosphatase, non-receptor type 12	AL079909	133,28	14,99	60,41	0,79	−2,21	0,03174
244757_at	Cytochrome P450, family 2, subfamily R, polypeptide 1	AI692525	98,81	17,21	44,57	21,09	−2,22	0,01478
204296_at	gb:NM_021196.1	NM_021196	109,35	26,51	49,24	7,95	−2,22	0,04929
224250_s_at	SECIS binding protein 2	BC001189	261,83	60,16	117,72	9,62	−2,22	0,04101
212952_at	Calreticulin	AA910371	1511,5	396,05	675,96	133,87	−2,24	0,03178
243993_at	PCTAIRE protein kinase 2	AA436887	393,92	77,35	176,13	6,16	−2,24	0,03576
232118_at	Chromosome 20 open reading frame 155	R33735	298,56	7,84	132,41	47,33	−2,25	0,03847
202028_s_at	gb:BC000603.1	BC000603	2150,5	446,40	939,35	211,88	−2,29	0,01471
209207_s_at	SEC22 vesicle trafficking protein-like 1 (S. cerevisiae)	BC001364	705,55	75,30	307,12	77,55	−2,3	0,00335
242560_at	Fanconi anemia, complementation group D2	AA579890	121,37	27,26	52,58	26,19	−2,31	0,04905
226085_at	Chromobox homolog 5 (HP1 alpha homolog, Drosophila)	AA181060	674,11	177,98	288,47	107,20	−2,34	0,0154
206438_x_at	hypothetical protein FLJ12975	NM_024809	488,81	19,84	207,71	74,71	−2,35	0,03745
1560402_at	growth arrest-specific 5	BF336936	430,65	47,57	182,35	90,17	−2,36	0,01151
225116_at	Homeodomain interacting protein kinase 2	AW300045	539,53	90,42	228,41	79,96	−2,36	0,01139
214395_x_at	Eukaryotic translation elongation factor 1 delta (guanine nucleotide exchange protein)	AI335509	194,14	37,67	82,06	44,72	−2,37	0,03623
1558019_at	Dystonin	BC020911	327,5	63,55	137,33	30,17	−2,38	0,01177
1564072_at	gb:AK025690.1	AK025690	1020,1	152,50	428,9	82,66	−2,38	0,03267
226643_s_at	NudC domain containing 2	AI291200	373,57	27,38	156,7	79,47	−2,38	0,02316
231393_x_at	Zinc finger protein 297B	AW237165	158,76	22,10	66,09	26,02	−2,4	0,03737
231370_at	Protein phosphatase 1A (formerly 2C), magnesium-dependent, alpha isoform	AI701170	264,16	54,30	108,58	26,09	−2,43	0,01432
1563283_at	Homo sapiens, clone IMAGE:4828909, mRNA	BG718722	843,46	98,58	345,82	109,30	−2,44	0,00876
213426_s_at	caveolin 2	AA150110	470,58	114,98	189,36	102,20	−2,49	0,0063
213734_at	WD repeat and SOCS box-containing 2	BG260658	740,32	176,82	296,21	80,29	−2,5	0,01662
229165_at	Mitochondrial ribosomal protein L12	BF433010	162,11	10,83	64,37	23,71	−2,52	0,03196
202648_at	ribosomal protein S19	BC000023	529,75	51,94	209,85	38,50	−2,52	0,00463
220839_at	methyltransferase like 5	NM_014168	237,93	77,58	93,29	36,86	−2,55	0,04023
242485_at	PTK2 protein tyrosine kinase 2	AW178807	166,68	31,29	64,65	10,50	−2,58	0,0348
230820_at	SMAD specific E3 ubiquitin protein ligase 2	BF111169	1460,4	212,19	549,25	150,26	−2,66	0,00218
222027_at	Nuclear ubiquitous casein kinase and cyclin-dependent kinase substrate	AW515443	111,81	7,14	41,65	10,47	−2,68	0,03024
212649_at	gb:AL079292.1	AL079292	202,52	44,29	75,22	39,30	−2,69	0,02723
228477_at	Hypothetical protein FLJ10154	R53084	1465,2	151,71	541,96	142,16	−2,7	0,00166
239859_x_at	ATP synthase, H+ transporting, mitochondrial F0 complex, subunit s (factor B)	AW140122	93,77	4,18	33,72	15,53	−2,78	0,04668
210758_at	PC4 and SFRS1 interacting protein 1	AF098482	444,61	36,70	156,69	37,48	−2,84	0,02343
1557830_at	Cyclin J	AW063658	367,31	19,61	126,36	23,00	−2,91	0,00916
1559521_at	MRNA full length insert cDNA clone EUROIMAGE 29093	AL355741	258,03	21,91	84,38	45,58	−3,06	0,02554
200908_s_at	ribosomal protein, large P2	BC005354	575,68	24,55	187,15	108,90	−3,08	0,02087
224375_at	gb:AF271776.1	AF271776	795,28	79,08	248,14	145,83	−3,2	0,01506
233204_at	Hypothetical protein MGC40405	AA115105	107,54	17,25	30,82	10,88	−3,49	0,02822
230084_at	solute carrier family 30 (zinc transporter), member 2	BF510698	814,56	203,67	232,49	146,26	−3,5	0,01187
232351_at	CDNA FLJ12246 fis, clone MAMMA1001343	AK022308	158,29	39,36	45,15	10,32	−3,51	0,03121
243885_x_at	Latexin	AA526937	92,06	29,95	26,17	11,58	−3,52	0,03092
241617_x_at	gb:BE961949	BE961949	93,34	23,47	25,35	11,78	−3,68	0,02206
214313_s_at	Eukaryotic translation initiation factor 5B	BE138647	113,14	16,37	29,69	14,69	−3,81	0,02274
1553749_at	hypothetical protein MGC33371	NM_144664	194,53	71,06	50,5	27,11	−3,85	0,04744
1553575_at	gb:NM_173714.1	NM_173714	440,06	81,16	108,49	27,98	−4,06	0,01314
220787_at	gb:NM_018629.1	NM_018629	72,95	6,02	17,61	5,48	−4,14	0,01972
213813_x_at	gb:AI345238	AI345238	232,05	35,44	55	23,58	−4,22	0,00713
233047_at	hypothetical protein LOC90167	AL161984	451,93	128,65	84,7	14,81	−5,34	0,03067

### Comparison of Gene Expression Profiles by Hierarchical Clusters and Principal Component Analysis (PCA)

**Figure 1:**
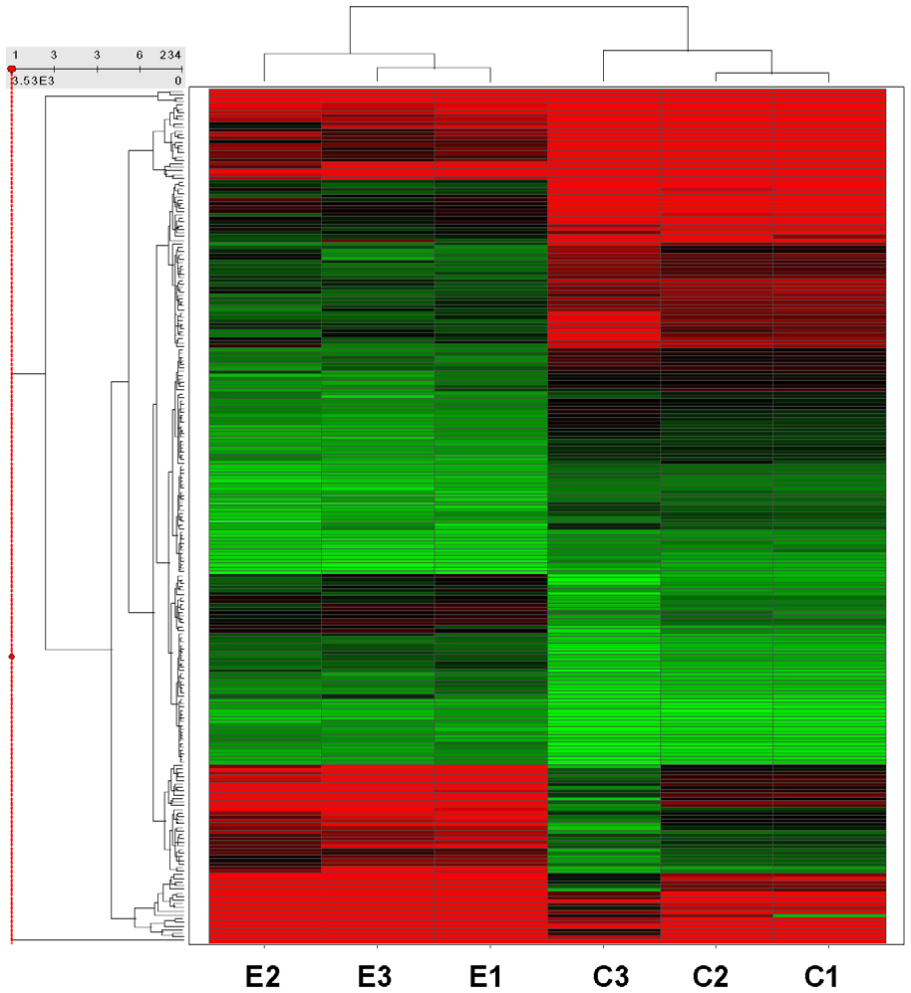
Supervised hierarchical cluster of HUVEC gene expression changes in response to estradiol. 263 probe sets of genes significantly regulated by greater than 1.8-fold change were used for 2D hierarchical clustering. Each row represents an individual probe set, and each column represents a pool of cells (C1, C2 and C3: control samples; E1, E2 and E3: estradiol-treated samples). 129 up- (red) *or* 134 down- (green) were regulated (P value<0.05).

Hierarchical Clusters were used to analyze the expression profile of the different samples (Figure 1). Results identified broad similarities among arrays hybridized with the mRNA of control cells or among arrays hybridized with mRNA of cells treated with estradiol. Even though the overall signal patterns found on the mRNA hybridized arrays were similar, a small subset of regions show differential expression signals between the mRNA of control cells and mRNA of cells treated with estradiol.

**Figure 2:**
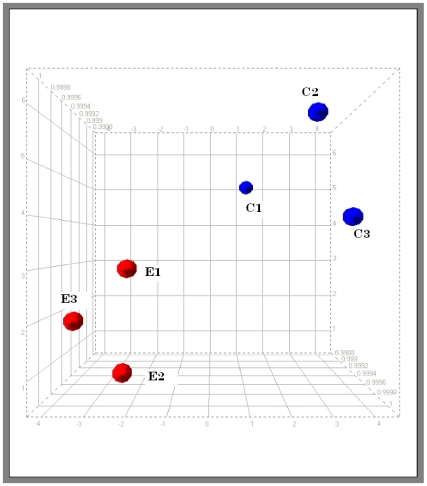
Supervised principal component analysis (PCA). Microarray hybridizations were performed using total RNA from HUVEC exposed to 1 nmol/L estradiol for 24 h. The gene expression profiles of 3 pools of control cells (blue) and 3 pools of cells treated with estradiol 1 nmol/L (red) were compared using PCA. The three-dimensional (3D) plot view of gene expression data (including all probe sets on U133 Plus 2.0 GeneChip) is shown, with respect to their correlation to the first three principal components.

PCA was applied to establish the interrelationships among the samples used in our study. By visualizing projections of these components in low-dimensional spaces, samples were grouped, reflecting underlying patterns in their gene expression profiles. Figure 2 depicts the PCA with the six pools clearly separated into two sets, one set with three control samples, and the other set with three estradiol-treated samples.

### Functional Categorization of Genes

HUVEC genes regulated by estradiol were organized by function to better understand their profile. This classification showed that estradiol regulated a great number of genes mainly associated with biological processes that include Cellular Growth and Proliferation; Cell-to-cell Signaling; Cellular Assembly and Organization; Cellular Compromise; Cellular Movement and Cell Death, as shown in Table 3 (online supporting information). The Cardiovascular System Development and Function also appears as one of the main regulated. Genes with a role in Lipid and Carbohydrate Metabolism, Cell Signaling, Endocrine System Disorders or Metabolic Disease appear to be significantly regulated by estrogens as well. Among these biological processes, the greater part of molecules induced by estradiol in HUVEC is related to growth of cells (47 molecules), cell death (47 molecules) and apoptosis (39 molecules), cell movement (32 molecules), growth of eukaryotic cells (25 molecules) adhesion cells (22 molecules), colony formation (16 molecules), development of blood vessels (15 molecules), cell surface receptor linked signal transduction (14 molecules) and angiogenesis (10 molecules).

**Table 3:** Functional analysis of differentially expressed genes in estradiol-treated HUVEC.

Category	Process Annotation	Significance	Molecules
**Cellular Growth and Proliferation**	growth of cells	0,0000	47
	growth of kidney cell lines	0,0102	4
	growth of fibrosarcoma cell lines	0,0103	2
	growth of eukaryotic cells	0,0111	25
	arrest in growth of pre-B lymphocytes	0,0143	1
	growth of colon cell lines	0,0143	1
	growth of hepatoma cell lines	0,0147	3
	growth of cell lines	0,0156	20
	growth of embryonic cell lines	0,0158	3
	growth of lymphoma cell lines	0,0170	3
	growth of melanoma cells	0,0215	2
	growth of leukemia cell lines	0,0218	4
	arrest in growth of cells	0,0266	6
	colony formation	0,0002	16
	colony formation of cells	0,0002	16
	colony formation of eukaryotic cells	0,0003	15
	colony formation of tumor cell lines	0,0004	10
	colony formation of carcinoma cell lines	0,0020	3
	colony formation of cell lines	0,0020	11
	colony formation of red blood cells	0,0059	3
	colony formation of blood cells	0,0062	5
	colony formation of lung cancer cell lines	0,0073	3
	colony formation of bone cancer cell lines	0,0086	2
	colony formation of leukemia cell lines	0,0086	2
	colony formation of connective tissue cells	0,0098	3
	colony formation of erythroid cells	0,0103	2
	colony formation of stromal cells	0,0143	1
	colony formation of erythroid cell lines	0,0144	2
	colony formation of bone marrow cells	0,0149	4
	colony formation of lymphatic system cells	0,0165	4
	colony formation of myeloid cells	0,0251	3
	colony formation of prostate cancer cell lines	0,0270	2
	proliferation of endothelial cells	0,0015	7
	arrest in proliferation of bone cancer cell lines	0,0143	1
	proliferation of granulocytes	0,0215	2
	formation of osteoclast-like cells	0,0123	2
	formation of epithelial cell lines	0,0143	1
	formation of lung cancer cell lines	0,0143	1
	formation of macrophages	0,0215	2
	formation of blood cells	0,0266	3
	induction of mesenchymal cells	0,0143	1
	inhibition of endothelial cell lines	0,0143	1
	inhibition of endothelial cells	0,0143	1
	inhibition of ovarian cancer cell lines	0,0143	1
	inhibition of smooth muscle cells	0,0143	1
	stimulation of progenitor cells	0,0143	1
	suppression of fibroblast cell lines	0,0143	1
	suppression of lung cell lines	0,0143	1
	expansion of hematopoietic progenitor cells	0,0190	2
**Cell-To-Cell Signaling and Interaction**	binding of stem cells	0,0002	2
	binding of female germ cells	0,0054	2
	binding of embryonic stem cells	0,0143	1
	binding of stromal cell lines	0,0143	1
	binding of sperm	0,0242	2
	adhesion of cells	0,0012	22
	adhesion of tumor cell lines	0,0176	7
	attachment of brain cancer cell lines	0,0020	2
	attachment of tumor cell lines	0,0073	3
	attachment of intestinal cell lines	0,0143	1
	attachment of spermatids	0,0143	1
	attachment of spermatocytes	0,0143	1
	attachment of cell lines	0,0170	3
	attachment of eukaryotic cells	0,0280	4
	accumulation of focal adhesions	0,0143	1
	activation of carcinoma cell lines	0,0143	1
	contact growth inhibition of fibrosarcoma cell lines	0,0143	1
	development of intercalated disks	0,0143	1
	disassembly of adherens junctions	0,0143	1
	induction of mesenchymal cells	0,0143	1
	maintenance of focal adhesions	0,0143	1
	production of cell-associated matrix	0,0143	1
	response of breast cancer cell lines	0,0143	1
	sensitization of leukocyte cell lines	0,0143	1
	stimulation of progenitor cells	0,0143	1
	suppression of fibroblast cell lines	0,0143	1
	suppression of lung cell lines	0,0143	1
**Cellular Compromise**	fragmentation of vesicles	0,0002	2
	fragmentation of hepatocytes	0,0143	1
	degeneration of epithelial cells	0,0012	2
	degeneration of cells	0,0061	6
	degeneration of keratinocytes	0,0143	1
	degeneration of renal tubular epithelial cells	0,0143	1
	shrinkage of cells	0,0086	2
	depletion of podocytes	0,0143	1
	disassembly of adherens junctions	0,0143	1
	disruption of PML nuclear bodies	0,0143	1
	disruption of spindle pole	0,0143	1
**Cardiovascular System Development and Function**	development of vascular tissue	0,0002	3
	development of blood vessel	0,0005	15
	proliferation of endothelial cells	0,0015	7
	cell flattening of endothelial cells	0,0143	1
	concentration of blood vessel	0,0143	1
	inhibition of endothelial cell lines	0,0143	1
	inhibition of endothelial cells	0,0143	1
	length of endothelial tube	0,0143	1
	migration of cardiomyocytes	0,0143	1
	muscularization of pulmonary artery	0,0143	1
	thickness of right ventricle of heart	0,0143	1
	angiogenesis	0,0182	10
	angiogenesis of tumor	0,0251	3
	vasculogenesis	0,0236	3
**Carbohydrate Metabolism**	modification of polysaccharide	0,0002	4
	modification of N-glycan	0,0003	3
	modification of carbohydrate	0,0181	4
	metabolism of glucose-6-phosphate	0,0012	2
	moiety attachment of polysaccharide	0,0041	2
	moiety attachment of carbohydrate	0,0166	2
	processing of N-glycan	0,0069	2
	galactosylation of N-glycan	0,0143	1
	utilization of glucose-6-phosphate	0,0143	1
**Skeletal and Muscular System Development and Function**	area of muscle cells	0,0006	3
	formation of osteoclast-like cells	0,0123	2
	development of intercalated disks	0,0143	1
	development of tracheal ring	0,0143	1
	inhibition of smooth muscle cells	0,0143	1
	length of skeleton	0,0143	1
	migration of cardiomyocytes	0,0143	1
	morphology of skeletal muscle	0,0143	1
	muscularization of pulmonary artery	0,0143	1
	size of medullary cavity	0,0143	1
	myogenesis of organism	0,0215	2
	differentiation of bone cell lines	0,0280	4
**Cellular Assembly and Organization**	accumulation of actin filaments	0,0012	2
	accumulation of filaments	0,0054	2
	accumulation of focal adhesions	0,0143	1
	assembly of vesicles	0,0030	2
	assembly of actin filaments	0,0090	4
	cross-linkage of actin filaments	0,0030	2
	cross-linkage of microfilaments	0,0143	1
	biogenesis of actin cytoskeleton	0,0065	5
	biogenesis of mitochondria	0,0144	2
	biogenesis of cytoskeleton	0,0167	6
	association of actin cytoskeleton	0,0143	1
	deposition of collagen fibrils	0,0143	1
	deposition of reticulin fiber networks	0,0143	1
	detachment of desmin filament	0,0143	1
	development of intercalated disks	0,0143	1
	development of mitochondria	0,0190	2
	disruption of PML nuclear bodies	0,0143	1
	disruption of spindle pole	0,0143	1
	immobilization of actin filaments	0,0143	1
	maturation of olfactory glomeruli	0,0143	1
	organization of cell cortex	0,0143	1
	organization of microtubules	0,0242	2
	polymerization of actin stress fibers	0,0143	1
	production of cell-associated matrix	0,0143	1
	quantity of mitochondrial contact sites	0,0143	1
	quantity of multivesicular bodies	0,0143	1
	formation of actin filaments	0,0145	7
	formation of filaments	0,0254	8
	formation of axons	0,0270	2
	stabilization of filaments	0,0251	3
	reorganization of actin	0,0270	2
**Cellular Movement**	chemotaxis of lymphocytes	0,0025	6
	chemotaxis of mononuclear leukocytes	0,0030	7
	chemotaxis of natural killer cells	0,0042	3
	chemotaxis of T lymphocytes	0,0134	4
	chemotaxis of leukocytes	0,0227	7
	chemotaxis of blood cells	0,0275	7
	migration of dermal fibroblasts	0,0030	2
	migration of trophoblast cells	0,0054	2
	migration of embryonic cells	0,0127	4
	migration of cardiomyocytes	0,0143	1
	migration of endodermal cells	0,0143	1
	homing of lymphocytes	0,0041	6
	homing of mononuclear leukocytes	0,0045	7
	homing of T lymphocytes	0,0165	4
	cell movement	0,0048	32
	cell movement of dermal fibroblasts	0,0069	2
	cell movement of natural killer cells	0,0098	3
	cell movement of embryonic cells	0,0141	4
	cell movement of lymphocytes	0,0147	8
	cell movement of mononuclear leukocytes	0,0177	9
	release of cells	0,0086	2
	infiltration of hairy leukemia cells	0,0143	1
	locomotion of neutrophils	0,0143	1
	scattering of pancreatic cancer cells	0,0143	1
	scattering of squamous carcinoma cells	0,0143	1
	translocation of spermatids	0,0143	1
	haptotaxis of tumor cell lines	0,0144	2
**Cell Death**	cell death of fibroblast cell lines	0,0002	13
	cell death of cell lines	0,0019	32
	cell death of kidney cell lines	0,0019	9
	cell death of prostate cell lines	0,0041	2
	cell death of eukaryotic cells	0,0051	40
	cell death	0,0052	47
	cell death of embryonic cell lines	0,0067	7
	cell death of neuroblastoma cell lines	0,0109	5
	cell death of thyroid cells	0,0123	2
	cell death of muscle cells	0,0133	6
	cell death of endothelial cell lines	0,0165	4
	cell death of epithelial cell lines	0,0185	7
	delay in cell death of cell lines	0,0190	2
	cell death of eosinophils	0,0215	2
	cell death of nervous tissue cell lines	0,0266	3
	cell death of splenocytes	0,0270	2
	regeneration of blood cells	0,0012	2
	regeneration of blood platelets	0,0143	1
	regeneration of hematopoietic progenitor cells	0,0143	1
	regeneration of red blood cells	0,0143	1
	apoptosis of kidney cell lines	0,0014	8
	apoptosis of prostate cell lines	0,0020	2
	apoptosis of embryonic cell lines	0,0066	6
	delay in apoptosis of tumor cell lines	0,0103	2
	apoptosis of thyroid cells	0,0123	2
	apoptosis of fibroblast cell lines	0,0123	8
	apoptosis of granulocyte-macrophage progenitor cells	0,0143	1
	apoptosis of liver cells	0,0157	4
	delay in apoptosis of cell lines	0,0190	2
	apoptosis of epithelial cell lines	0,0195	6
	apoptosis	0,0200	39
	apoptosis of eosinophils	0,0215	2
	apoptosis of splenocytes	0,0215	2
	apoptosis of muscle cells	0,0227	5
	apoptosis of hippocampal cells	0,0242	2
	apoptosis of cerebral cortex cells	0,0266	3
	apoptosis of stomach cancer cell lines	0,0270	2
	colony survival of eukaryotic cells	0,0047	3
	colony survival	0,0059	3
	colony survival of cells	0,0059	3
	colony survival of lymphoma cell lines	0,0143	1
	cell viability of neuroblastoma cell lines	0,0086	2
	survival of endocrine cells	0,0103	2
	survival of germ cells	0,0123	2
	survival of gonadal cells	0,0144	2
	survival of pheochromocytoma cell lines	0,0242	2
	survival of hematopoietic cells	0,0270	2
**Lipid Metabolism**	biosynthesis of phosphatidylinositol	0,0041	2
	biosynthesis of phosphatidic acid	0,0042	3
	biosynthesis of phospholipid	0,0116	3
	biosynthesis of dolichol monophosphate mannose	0,0143	1
	binding of 1-alpha, 25-dihydroxy vitamin D3	0,0143	1
	formation of 5(S)-HETE	0,0143	1
	production of 25-hydroxy-vitamin D3	0,0143	1
	production of cholecalciferol	0,0143	1
	reduction of ceramide	0,0143	1
	utilization of triacylglycerol	0,0143	1
**Cardiovascular Disease**	hypoplasia of myocardium	0,0054	2
	fibrosis of portal artery	0,0143	1
	muscular dystrophy of cardiac muscle	0,0143	1
	tetralogy of Fallot of mice	0,0143	1
	cell death of endothelial cell lines	0,0165	4
	cardiovascular disorder of heart	0,0195	3
**Cell Signaling**	cell surface receptor linked signal transduction	0,0063	14
	suppression of nitric oxide	0,0143	1
	androgen receptor signaling pathway	0,0166	2
**Metabolic Disease**	amyloidosis	0,0106	3
	familial partial lipodystrophy type 2	0,0143	1
	pseudohypoparathyroidism, type 1B	0,0143	1
**Endocrine System Disorders**	apoptosis of thyroid cells	0,0123	2
	cell death of thyroid cells	0,0123	2
	differentiation of pancreatic cancer cells	0,0143	1
	McCune-Albright syndrome	0,0143	1
	pseudohypoparathyroidism, type 1B	0,0143	1
	scattering of pancreatic cancer cells	0,0143	1
	spontaneous autoimmune thyroiditis of mice	0,0143	1
	survival of pheochromocytoma cell lines	0,0242	2
**Free Radical Scavenging**	production of reactive oxygen species	0,0243	5

**Figure 3:**
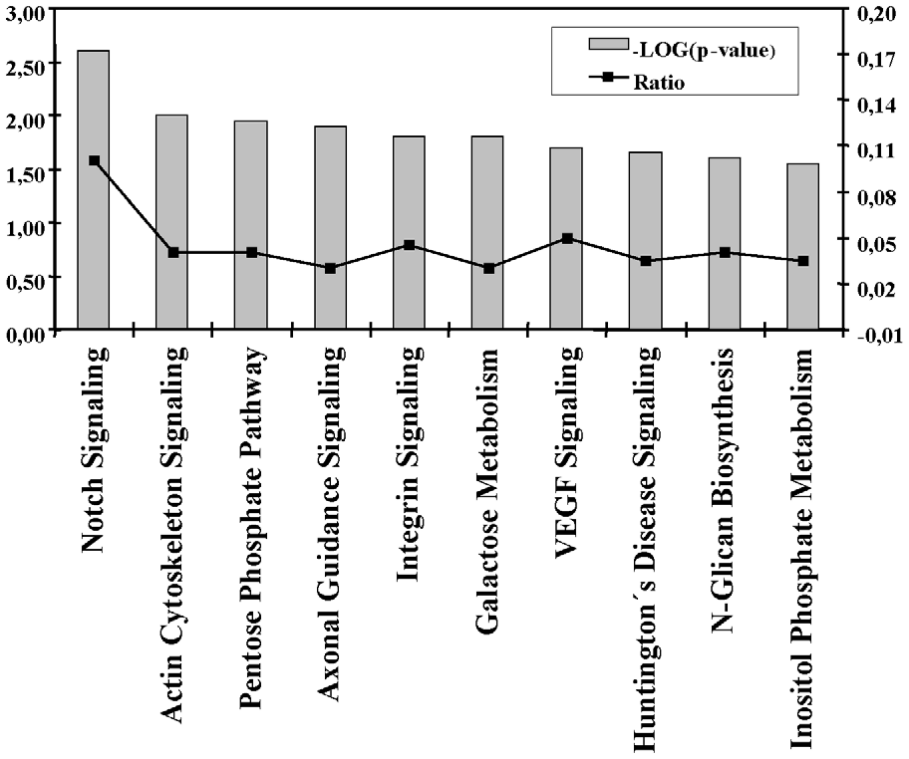
Top ten signaling and metabolic pathways regulated by estradiol. For the functional categorization of genes, Fischer's exact test was used to calculate a p value (shown as bars) determining the probability that each biological function assigned to the network is due to chance alone. The ratio (shown as squares) represents the number of differentially expressed genes in a given pathway divided by total number of genes that make up that canonical pathway.

The functional characterization of data are presented in Figure 3, which lists top ten canonical pathways regulated by estrogen across both tissue types. The top five canonical pathways based on their significance (P value) included Notch Signaling, Actin Cytoskeleton Signaling, Pentose Phosphate Pathway, Axonal Guidance Signaling and Integrin Signaling. Genes included in each group of the top ten signaling pathways presented in Figure 3 are listed in Table 4.

**Table 4:** Significant genes included in the top ten canonical pathways presented in Figure 3.

Cannonical pathway	Significant genes included in each group of the top ten canonical pathways
Notch signaling	FURIN, JAG2, NOTCH4, RFNG
Actin cytoskeleton signaling	ACTN4, ARHGEF1, ARHGEF12,GRLF1, GSN, IQGAP3,MAPK3, MYH16, MYL9, PAK4, PDGFA, PIP5K1C, RAC2
Pentose phosphate pathway	G6PD, GPI, PFKL, PGLS, PRPS2
Axonal guidance signaling	ADAM15, AKT1, ARHGEF12, GNA11, GNB2, MAPK3, MYL9,PAK4, PDGFA, PLXNB2, PPP3CA, RAC2, SEMA6B
Integrin signaling	ACTN4, AKT1, ARF3, ARF6, ITGA5, MAPK3, PAK4, PARVB, RAC2, RHOC, TNK2
Galactose metabolism	GALT, UGALT, UGT2
VEGF signaling	ARNT, PI3K, ACTC, BCL-XL
Huntington's disease signaling	AKT1, ARFIP2, CTSD, DCTN1, GNA11, GNB2, HDAC7A, POLR2L, SNCA, TGM2, UBE2S
N-glycan biosynthesis	B4GALT5, DPM3, MAN1B1, MGAT4B
Inositol phosphate metabolism	IP3K, PI4K, PI3K

**Figure 4:**
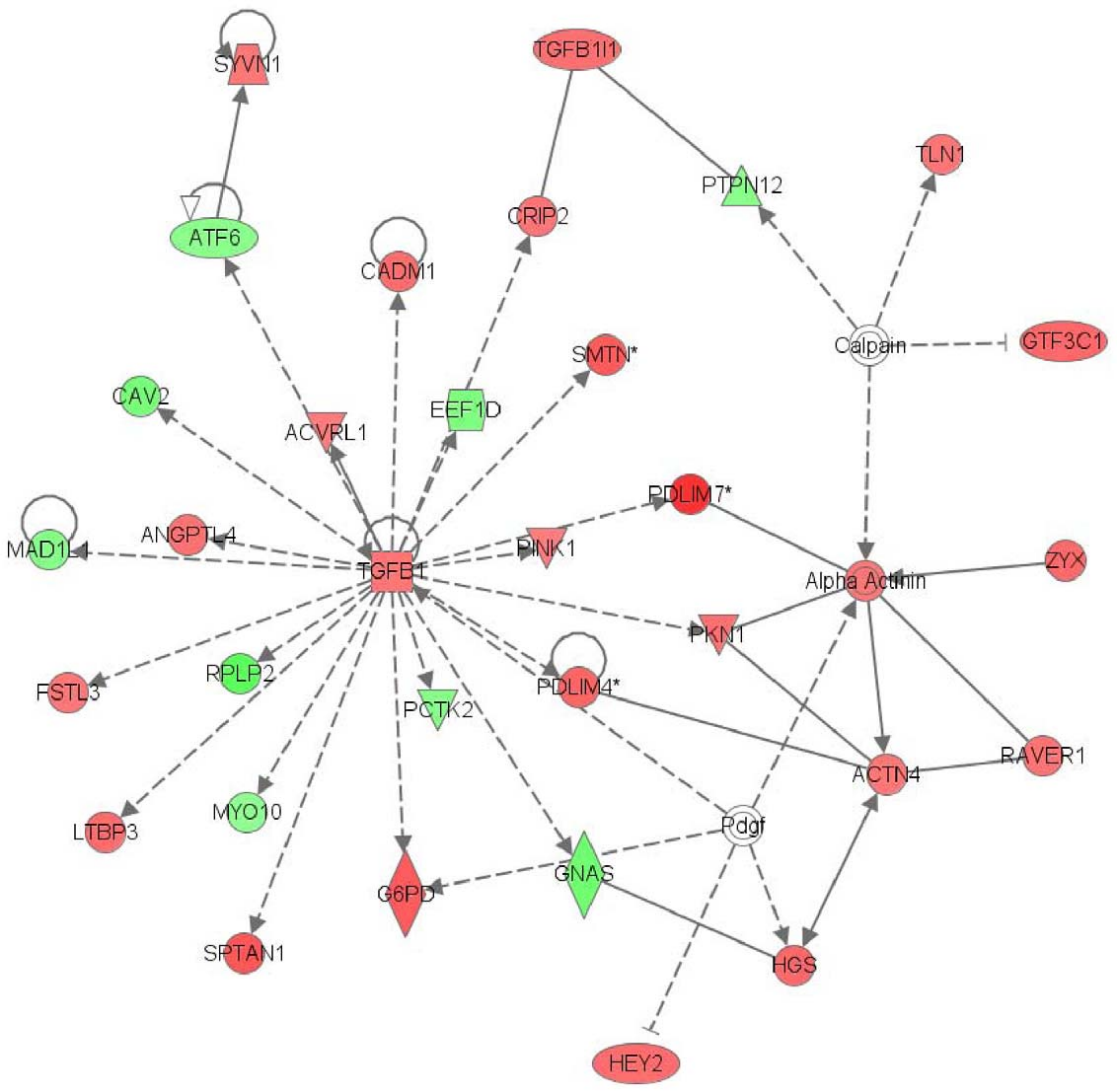
The most significant network regulated by estradiol is centered on TGFB1. Networks of genes were algorithmically generated with the IPA software based on their connectivity and assigned a score. The intensity of the node color indicates the degree of up- (red) or down- (green) regulation. A continuous line means a direct relationship between the two genes, whereas a discontinuous line indicates an indirect association. The most significant network regulated by estradiol includes 33 genes with an assigned score of 62 and is centered on TGFB1.

When the IPA software was used to analyze the probe sets there were 26 significant regulatory networks (score>2), of which 5 were highly significant (score>20). The number one ranked network (score = 62, focus molecules = 33) (Figure 4) is associated with Cardiovascular System Development and Function, Cellular Growth and Proliferation and Cell Morphology. Transforming Growth Factor beta-1 (TGFB1) plays a central role in the formation of this network. Top functions of the other four highly significant networks are associated with Cellular Compromise, Cellular Movement, Hematological System Development and Function, Lipid Metabolism, Molecular Transport and Small Molecule Biochemistry.

### Microarray Analysis Verification

To validate the HUVEC gene expression changes induced by estradiol in the microarray analysis, QRT-PCR was performed in a separate series of follow-up studies of HUVEC exposed to different concentrations (0,01–100 nmol/L) of estradiol. Target genes were selected based upon their important cardiovascular functions and were genes encoding for DDAH1, DDAH2, PLA2G4A, PLA2G4B, COX1, COX2 and DHCR7.

**Figure 5:**
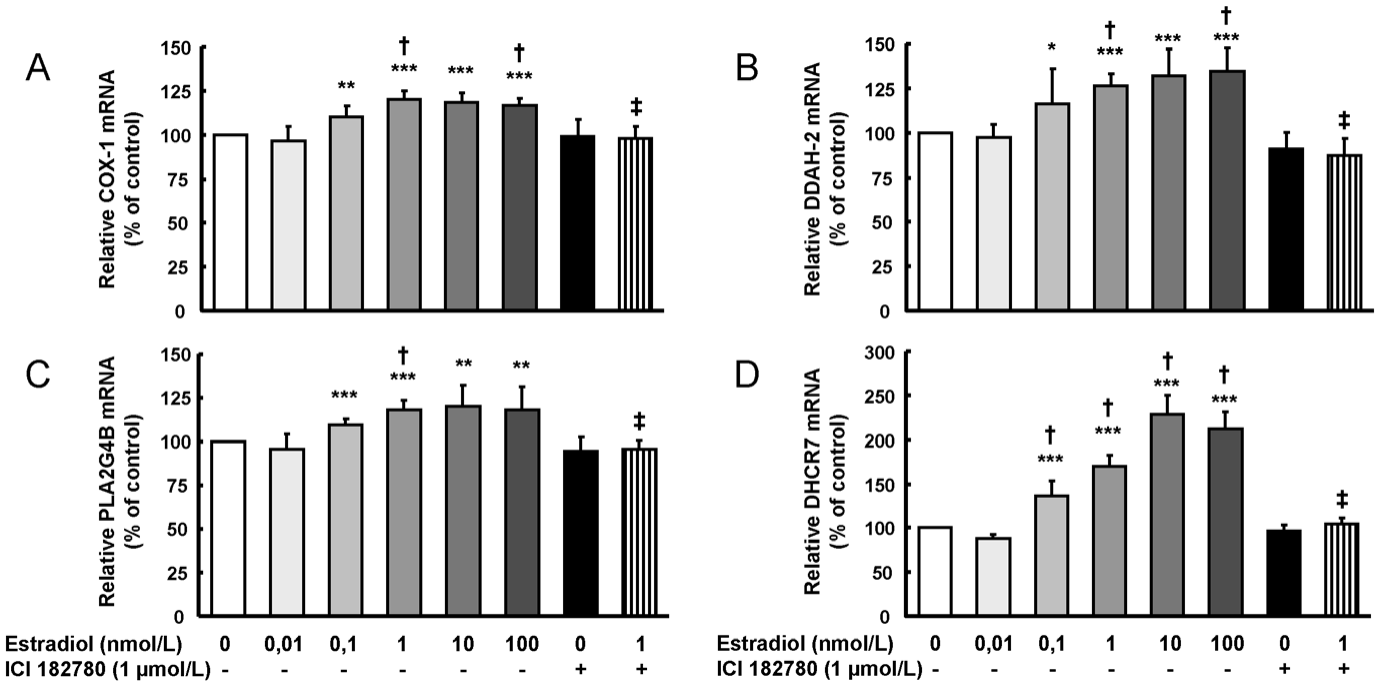
QRT-PCR confirms some estradiol up-regulated selected genes from the microarray analysis. HUVEC were exposed to different estradiol concentrations (0,01–100 nmol/L), and to 1 µmol/L ICI182780 alone or plus 1 nmol/L estradiol, and the relative expression of the genes was quantified: (A) COX1, (B) DDAH2, (C) PLA2G4B, and (D) DHCR7. Data are percentage of control values and are mean ± SEM of 5–19 values (4–6 different experiments). * p<0.05, ** p<0.01 or *** p<0.001 vs. control cells, † p<0.05 vs. 0.01 nmol/L estradiol, and ‡ p<0.05 vs. 1 nmol/L estradiol.

Estradiol dose-dependent increased mRNA expression of COX1, DDAH2, PLA2G4B, and DHCR7 (Figure 5). In all cases, the effect afforded by 1 nmol/L estradiol was significantly higher than that of 0,01 nmol/L estradiol. There were no differences between the effects on gene expression induced by the higher tested concentrations (1, 10 and 100 nmol/L), although in the case of DHCR7 the effect of 10 nmol/L was 34% higher than that of 1 nmol/l. The increased gene expressions induced by 1 nmol/L estradiol were similar to change levels obtained in the microarray analysis (probeset 205128_x_at for COX1, - fold change of 1.56 -p = 0.007-, probeset 202262_x_at for DDAH2 -fold change: 1.37, p = 0.045-, probeset 219095_at for PLA2G4B -fold change 1.56, p = 0.019-, and probeset 201790_s_at for DHCR7 -fold change 1.79, p = 0.005-).

**Figure 6:**
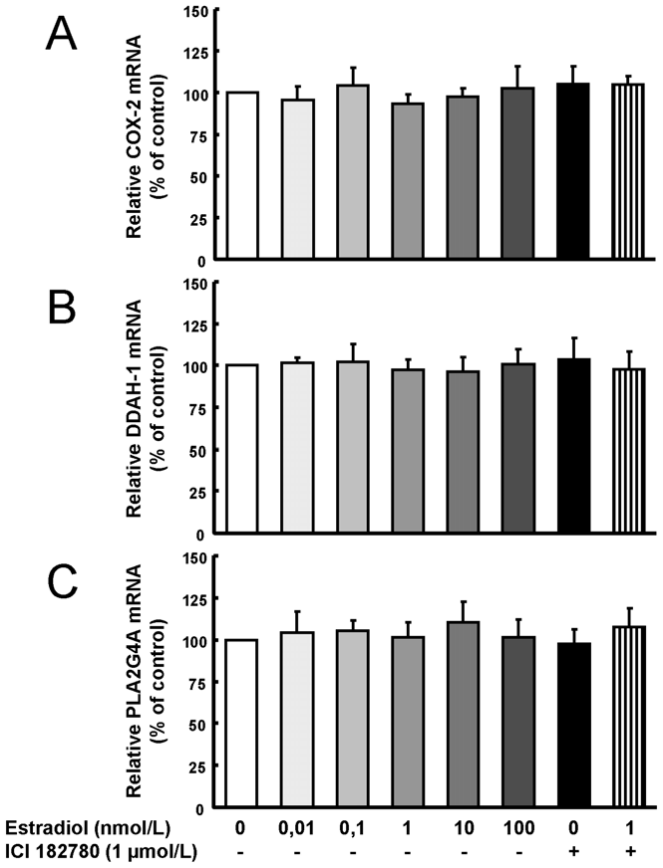
Unregulated genes in microarray analysis were also unchanged by QRT-PCR. HUVEC were exposed to different estradiol concentrations (0,01–100 nmol/L), and to 1 µmol/L ICI182780 alone or plus 1 nmol/L estradiol, for 24 hours. Total cellular RNA was extracted, and the relative expression of the genes was quantified using specific primers for (A) COX2, (B) DDAH1 and (C) PLA2G4A. The GADPH gene was used as control as described in Materials and Methods. Data are expressed as percentage of control values and are mean ± SEM of 5–17 values corresponding to 5 different experiments.

The mRNA expression of COX2, DDAH1 and PLA2G4A (Figure 6) remained unaltered under all the estradiol concentrations, as in the microarray analysis (probeset 204748_at for COX2 -fold change: −1.18, p = 0.541-, probeset 209094_at for DDAH1-fold change: −1.03, p = 0.743-, and probeset 210145_at for PLA2G4A -fold change −1.06, p = 0.570-).

**Figure 7:**
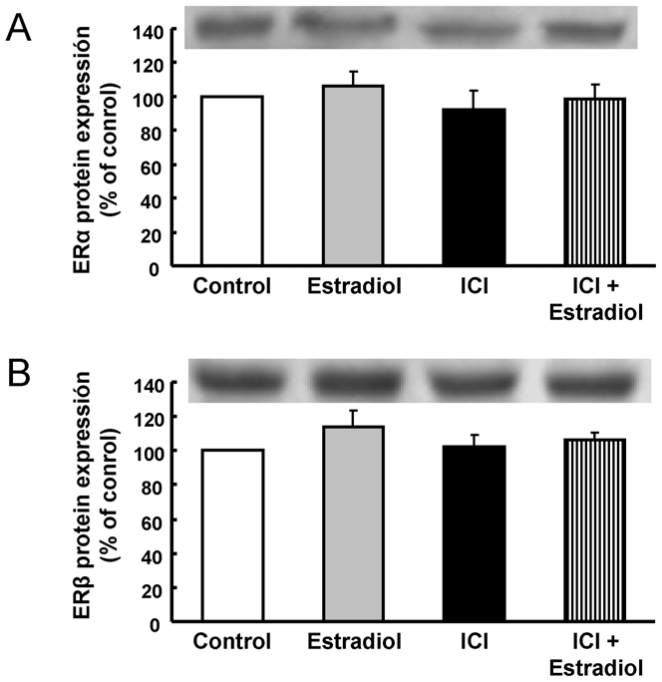
Estrogen receptor alpha and beta protein expression in HUVEC. Cells were exposed to 1 nmol/L estradiol with or without 1 µmol/L ICI182780 for 24 hours, and protein expression of (A) ERα and (B) ERβ were measured as stated in Materials and methods. A typical immunoblotting image and relative levels assessed by densitometry of bands of 66-kDa (ERα) or 56-kDa (ERβ) are presented. Data are percentage of control values and are mean ± SEM of 6 values (3 different experiments).

Estradiol genomic effects are mainly mediated through ERα and ERβ. HUVEC express both types of ER (Figure 7), and no changes in protein expression of both types of ER were observed after exposure to estradiol, ICI 182780, or estradiol plus ICI182780 (Figure 7). To study the role of ER on the effects induced by estradiol on gene expression, cells were exposed to the nonselective ER antagonist ICI182780. In cells exposed to different concentrations (0,01 – 10 µmol/L) of ICI 182780 alone, expression of the seven selected genes remained unaltered (Table 5), thus discarding a direct effect of ICI 182780 on gene profile. In cells coexposed to 1 nmol/L estradiol plus 1 µmol/L ICI182780 for 24 h, estradiol-induced effects on gene expression were completely abolished (Figure 5).

**Table 5:** Expression of selected genes from the microarray under different ICI 182780 concentrations.

ICI 182780 (µmol/L)	Gene expresión (% of control values)						
	DDAH-1	DDAH-2	PLA2G4A	PLA2G4B	COX-1	COX-2	DHCR7
0,01	96±9	102±3	95±8	99±6	104±11	99±10	98±4
0,1	110±9	102±11	95±7	100±7	101±7	96±9	102±7
1	95±8	108±12	108±8	106±7	107±6	101±9	105±10
10	101±10	101±7	105±12	106±11	100±3	98±16	102±9

Data are expressed as percentage of control values and are mean±SEM of 4–7 values corresponding to 2 different experiments.

**Figure 8:**
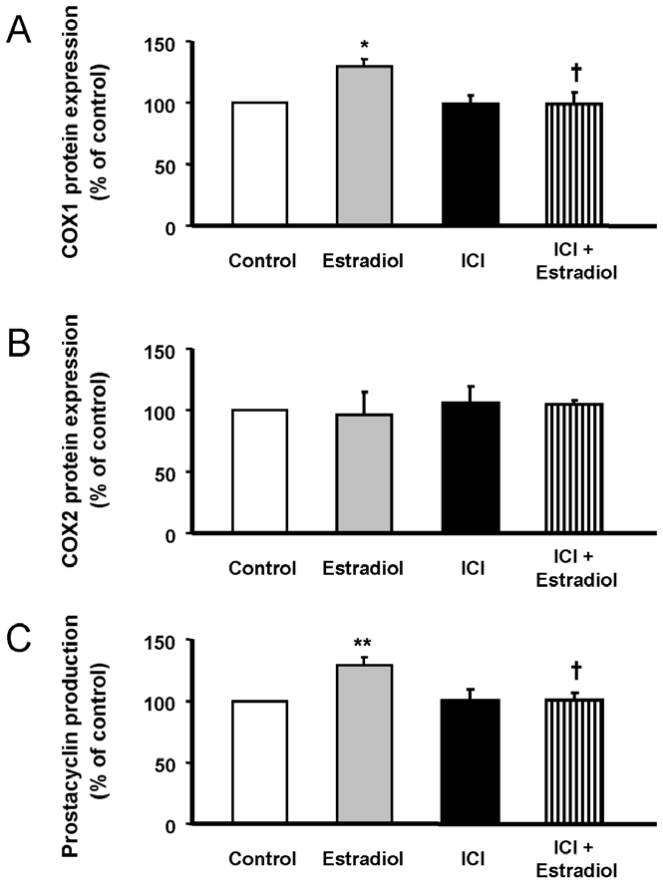
Estradiol up-regulated COX1 protein expression results in increased prostacyclin production through ER. HUVEC were exposed to 1 nmol/L estradiol with or without 1 µmol/L ICI182780, and protein expression of (A) COX1 and (B) COX2 and prostacyclin production (C) were measured as stated in Materials and methods. Data are percentage of control values and are mean ± SEM of 6–8 values (3–4 different experiments). * p<0.05 or ** p<0.01 vs. control cells, and † p<0.05 vs. estradiol-alone treated cells.

To further validate microarray data, COX1 and COX2 protein expression were analyzed by immunoblotting (Figure 8A and 8B). Estradiol increased COX1 protein expression up to 30 % of control values, whereas COX2 protein expression remained unchanged, in sharp agreement to data obtained from microarray analysis and QRT-PCR assays. Moreover, estradiol-induced COX1 up-regulation resulted in an increased production of prostacyclin, the main vasodilatory prostanoid regulated by COX activity (Figure 8C). These stimulatory effects of estradiol on prostacyclin synthesis pathway were mediated through ER activation, since were abolished in the presence of ICI 182780.

**Figure 9:**
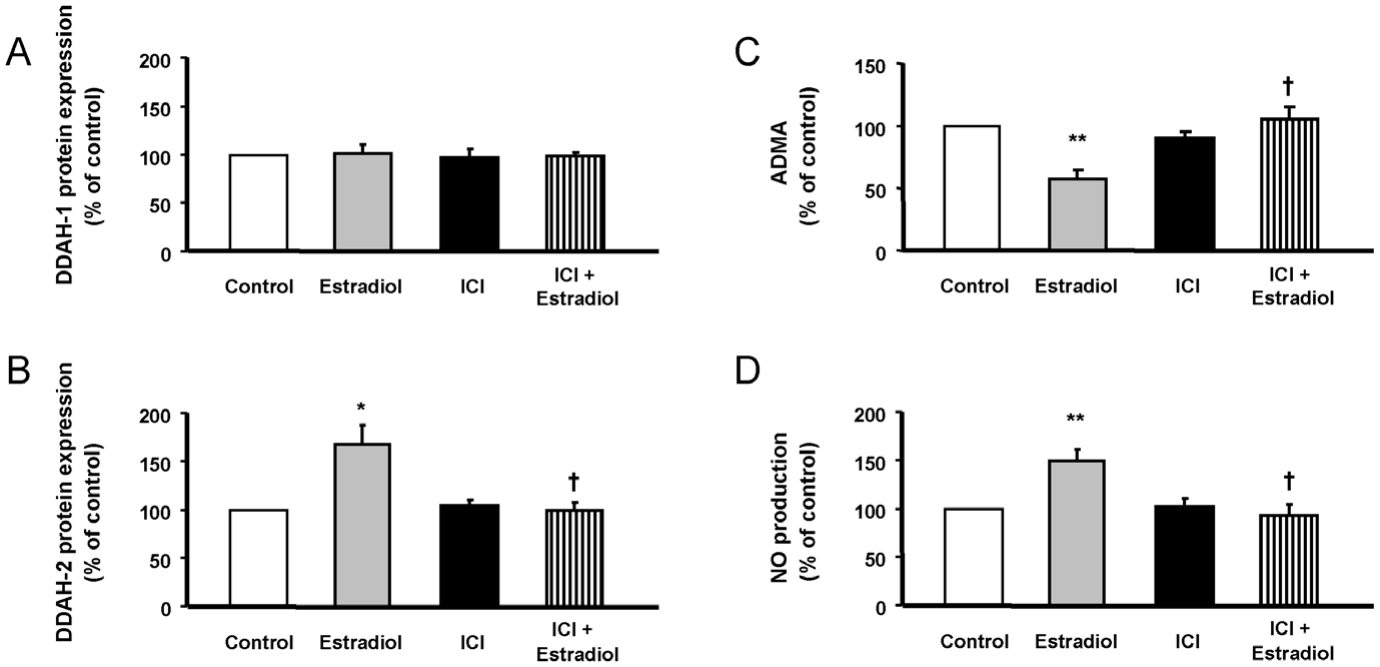
Estradiol up-regulated DDAH2 protein expression results in decreased ADMA production and increased NO release mediated by ER. HUVEC were exposed to 1 nmol/L estradiol with or without 1 µmol/L ICI182780, and protein expression of (A) DDAH1 and (B) DDAH2, along with (C) ADMA levels and NO production, were measured as stated in Materials and methods. Data are percentage of control values and are mean ± SEM of 9–12 values (4 different experiments). * p<0.05 or ** p<0.01 vs. control cells, and † p<0.05 vs. estradiol-alone treated cells.

In a similar way, estradiol-induced changes in the DDAH gene expression were correlated to similar changes in protein expression. DDAH2 protein expression was increased in the presence of estradiol, whereas DDAH1 remained unchanged (Figure 9A and 9B). *In vivo*, DDAH degrades most of ADMA [20], an endogenous inhibitor of NO synthase. The increased DDAH expression resulted in decreased ADMA production (Figure 9C), which in turn lead to an increased NO production after estradiol exposure (Figure 9D). The effects of estradiol on the DDAH-ADMA-NO pathways were mediated by ER, since were abolished in the presence of ICI 182780.

## Discussion

This study summarizes changes in complete gene expression in human endothelial cells exposed to estradiol. We have identified new genes that are up-regulated in endothelium by exposure to a physiological concentration of estradiol (1 nmol/L) for 24 hours, a time and a concentration selected according to previous work of our group [19]. We have identified 1886 genes differentially expressed. Taking advantage of ranked gene expression pathways, results have shown that pathways related to cellular growth and proliferation, cell-to-cell signaling and cellular organization, movement and death were among the most differentially expressed.

Canonical pathway analysis revealed Notch signaling as the most significant signaling pathway modulated by estradiol. Aberrant Notch signaling is implicated in carcinogenesis and tumor angiogenesis [21], and interestingly with human pathologies involving cardiovascular abnormalities [22]. Recently, it was reported that Notch pathway regulates cell-cell or cell-matrix interaction, contributing hence, to cell migration in situations of tissue remodeling [23]. Also, Notch1 has been implicated in the estradiol-induced increase in microvessel density *in vivo* and therefore in estradiol-increased tumor angiogenesis in MCF7 cells and HUVEC [24]. Our findings provide further support for the important role that Notch signaling pathway plays on endothelial effects of estradiol.

Estradiol has also important effects on other signaling pathways, mainly in Actin Cytoskeleton Signaling, Integrin Signaling, and Vascular Endothelial Growth Factor (VEGF) Signaling. These pathways exert important vascular actions, such as maintaining vascular integrity, regulating cell cycle, and promoting vasculogenesis. Moreover, four metabolic pathways are among the first 10 pathways significantly modulated by estradiol: Pentose Phosphate Pathway, Galactose Metabolism, N-Glycan Biosynthesis and Inositol Phosphate Metabolism. Some of these effects of estradiol have already been described. Estradiol, for instance, has already reported to increase HUVEC attachment to extracellular matrix proteins, mainly up-regulating surface expression of β1, α5 and α6 integrins [25]. Estradiol directly regulates the glucose-6-phosphate dehydrogenase (G6PDH) expression [26], the enzyme that directs glucose carbons into the pentose phosphate pathway. Moreover, estradiol-stimulated breast cancer cells have also increased pentose phosphate pathway activity, suggesting that this pathway is essential for estrogen-dependent cell proliferation [27]. Nevertheless, the majority of genes affected by estradiol treatment have been described for the first time in our results and our data open new approaches to discover unexplored estrogen-regulated pathways and new vascular actions.

The IPA software outlined the most changed pathways in the microarray data. Among them, TGFB1 plays a central role in the formation of the number-one-ranked network, which contains 33 genes (Figure 4) and is associated with other important cardiovascular networks, such as Cardiovascular System Development and Function, Cellular Growth and Proliferation and Cell Morphology. TGFB1 is a multifunctional peptide that controls proliferation, differentiation, and other functions in many cell types. In our study, TGFB1 was significantly up-regulated by estradiol as a main effect, supporting its important role in cardiovascular function. According to our results, estradiol exerts an important role in vessel assembly and stabilization through TGFB signaling pathways [28]. Moreover, TGFB pathway status determines the antiatherogenic effect of estradiol in apoE-/- hypercholesterolemic mice [29]. Furthermore, estradiol administration to postmenopausal women increases circulating levels of the active form of TGFB1 [30]. Altogether, these findings led to the conclusion that TGFB1 is one of the main targets of estradiol stimulation.

With the use of ICI 182780 in some experiments, our study demonstrates that activation of ER by E2 modifies the expression of several genes in HUVEC. In spite of some authors have found that HUVEC do not express ERα [31], other investigators have demonstrated the presence of both ERα and ERβ mRNA in HUVEC [32]. Data presented in Figure 7 demonstrate the expression of both ERα and ERβ protein in HUVEC, thus confirming previous reports [33], [34].

The extensive information gained from this first analysis has resulted in the collection of new data and new genes that provide other opportunities of study not explored so far, for example, the increased expression of the DHCR7 gene when HUVEC were exposed to different estradiol concentrations. This gene is responsible for the last step in cholesterol synthesis, and its inhibition results in hypocholesterolemia and accumulation of 7-dehidrocholesterol [35], while different mutations of this gene cause the Smith-Lemli-Opitz syndrome [36]. In our study, DHCR7 expression induced by 1 nmol/L estradiol was completely abolished in the presence of ICI182780 (Figure 5D). This is similar to the unique description of the relationship between this gene and estradiol, in which the expression of DHCR7 on human osteosarcoma cells was increased in response to estradiol through receptor beta [37].

Other cardiovascular-relevant genes confirmed the consistency of microarray data. Thus, the results are in accordance with similar effects observed in endothelial cells, both measuring the gene or the protein expression. COX are the rate-limiting step in the formation of vasoactive prostanoids, such as prostacyclin and thromboxane, from arachidonic acid [38]. Our results point to an estradiol-induced, dose-dependent gene expression, resulting in increased protein expression of COX-1 without effect on COX-2, which in turn resulted in increased prostacyclin production. These data, mediated through ER activity, have already been reported in some studies performed in ovine pulmonary artery endothelial cells [39], but not in others [40]. Related to COX-mediated prostanoid production, cytosolic phospholipase A2 activity is the initial step which liberates arachidonic acid from the cell membrane. In our study, PLA2G4B expression was reported to be dose-dependent increased by estradiol, while the main subtype PLA2G4A remained unaltered. Previous studies have reported an increase in cytosolic phospholipase A2 protein expression, without subtype differentiation, in ovine [41] and rat [42] uterine arteries exposed to estradiol.

ADMA is an analogue of arginine, which is synthesized endogenously and can act as inhibitor of nitric oxide synthase [20]. Both DDAH are responsible *in vivo* for ADMA degradation to citrulline and dimethylamine. According to the results obtained in the microarray analysis and confirmed by QRT-PCR and inmunoblotting, DDAH2 is increased in HUVEC exposed to different concentrations of estradiol, whereas DDAH1 remains unaltered. DDAH2, the main subtype in the cardiovascular system, has already been reported to be increased by estradiol in endothelium [19]. Moreover, the increased DDAH2 expression resulted in decreased ADMA concentration and therefore, increased NO release. Results of the present work further support that increased DDAH2 expression is dependent on ER-dependent genomic activity.

The strength of the current study is the careful design of the experiments and the use of sample pools which contribute to minimizing inter-individual variations. The average fold-change induced by estradiol is relatively low, but it should be taken into account that cells were exposed to estradiol concentrations that were within physiological levels in premenopausal women [43]. Moreover, fold-changes and number of up-regulated genes in our study were within the same range as that obtained in similar studies performed with higher estradiol concentrations (10–50 nmol/L) in different human breast cancer cell types [44].

Care should be taken determining clinically relevant consequences. Much *in vitro* and *in vivo* experimental data support a beneficial effect of estrogens on the cardiovascular system [6]. Observational studies have also consistently shown a benefit of hormone replacement therapy on cardiovascular disease, but some randomized studies have shown even some deleterious effects [6], [45]. New experimental approaches, such as the present study, should contribute to conciliate the divergences observed between clinical and experimental data.

In summary, our study generates a comprehensive estradiol-mediated gene expression profile in HUVEC and characterizes in detail the considerably different responses of control and estradiol-treated endothelial cells. The present study provides the first quantitative large-scale gene expression analysis of estradiol–stimulated human vascular endothelial cells. Identification of pathways regulated by estradiol may add to the knowledge base of how estradiol contributes to a wide range of biological processes. These results could lead to a deeper understanding of fundamental insights of pathophysiological mechanisms involved in cardiovascular diseases such as stroke or atherosclerosis at the level of gene expression and provide a foundation for the development of better therapeutic strategies for vascular disease.

## References

[1] Mendelsohn ME, Karas RH (2005). Molecular and cellular basis of cardiovascular gender differences.. Science.

[2] Barrett-Connor E, Grady D (1998). Hormone replacement therapy, heart disease, and other considerations.. Annu Rev Pub Health.

[3] Hulley S, Grady D, Bush T, Furberg C, Herrington D (1998). Randomized trial of estrogen plus progestin for secondary prevention of coronary heart disease in postmenopausal women. Heart and Estrogen/progestin Replacement Study (HERS) Research Group.. JAMA.

[4] Grady D, Herrington D, Bittner V, Blumenthal R, Davidson M (2002). Cardiovascular disease outcomes during 6.8 years of hormone therapy: Heart and Estrogen/progestin Replacement Study follow-up (HERS II).. JAMA.

[5] Rossouw JE, Anderson GL, Prentice RL, LaCroix AZ, Kooperberg C (2002). Risks and benefits of estrogen plus progestin in healthy postmenopausal women: principal results from the Women's Health Initiative randomized controlled trial.. JAMA.

[6] Turgeon JL, Carr MC, Maki PM, Mendelsohn ME, Wise PM (2006). Complex Actions of Sex Steroids in Adipose Tissue, the Cardiovascular System, and Brain: Insights from Basic Science and Clinical Studies.. Endocr Rev.

[7] Ross R (1999). Atherosclerosis: an inflammatory disease.. N Engl J Med.

[8] Mendelsohn ME (2000). Mechanisms of estrogen action in the cardiovascular system.. J Steroid Biochem Mol Biol.

[9] Mendelsohn ME (2002). Protective effects of estrogen on the cardiovascular system.. Am J Cardiol.

[10] Trevino V, Falciani F, Barrera-Saldana HA (2007). DNA microarrays: a powerful genomic tool for biomedical and clinical research.. Mol Med.

[11] Archacki SR, Wang QK (2006). Microarray analysis of cardiovascular diseases.. Methods Mol Med.

[12] Hiltunen MO, Tuomisto TT, Niemi M, Bräsen JH, Rissanen TT (2002). Changes in gene expression in atherosclerotic plaques analyzed using DNA array.. Atherosclerosis.

[13] Graham D, McBride MW, Gaasenbeek M, Gilday K, Beattie E (2007). Candidate genes that determine response to salt in the stroke-prone spontaneously hypertensive rat: congenic analysis.. Hypertension.

[14] Hermenegildo C, Oviedo PJ, Garcia-Perez MA, Tarin JJ, Cano A (2005). Effects of phytoestrogens genistein and daidzein on prostacyclin production by human endothelial cells.. J Pharmacol Exp Ther.

[15] Li C, Hung Wong W (2001). Model-based analysis of oligonucleotide arrays: model validation, design issues and standard error application.. Genome Biology.

[16] Edgar R, Domrachev M, Lash AE (2002). Gene Expression Omnibus: NCBI gene expression and hybridization array data repository.. Nucl Acids Res.

[17] Pfaffl MW, Horgan GW, Dempfle L (2002). Relative expression software tool (REST(C)) for group-wise comparison and statistical analysis of relative expression results in real-time PCR.. Nucl Acids Res.

[18] Lowry OH, Rosebrough NJ, Farr AL, Randall RJ (1951). Protein measurement with the Folin phenol reagent.. J Biol Chem.

[19] Monsalve E, Oviedo PJ, Garcia-Perez MA, Tarin JJ, Cano A (2007). Estradiol counteracts oxidized LDL-induced asymmetric dimethylarginine production by cultured human endothelial cells.. Cardiovasc Res.

[20] Vallance P, Leiper J (2004). Cardiovascular biology of the asymmetric dimethylarginine:dimethylarginine dimethylaminohydrolase pathway.. Arterioscler Thromb Vasc Biol.

[21] Leong KG, Karsan A (2006). Recent insights into the role of Notch signaling in tumorigenesis.. Blood.

[22] Uyttendaele H, Ho J, Rossant J, Kitajewski J (2001). Vascular patterning defects associated with expression of activated Notch4 in embryonic endothelium.. PNAS.

[23] Lindner V, Booth C, Prudovsky I, Small D, Maciag T (2001). Members of the Jagged/Notch gene families are expressed in injured arteries and regulate cell phenotype via alterations in cell matrix and cell-cell interaction.. Am J Pathol.

[24] Soares R, Balogh G, Guo S, Gartner F, Russo J (2004). Evidence for the Notch signaling pathway on the role of estrogen in angiogenesis.. Mol Endocrinol.

[25] Cid M, Esparza J, Schnaper H, Juan M, Yague J (1999). Estradiol enhances endothelial cell interactions with extracellular matrix proteins via an increase in integrin expression and function.. Angiogenesis.

[26] Thomas M, Bader C, Monet JD (1990). Sex steroid hormone modulation of NADPH pathways in MCF-7 cells.. Cancer Res.

[27] Forbes NS, Meadows AL, Clark DS, Blanch HW (2006). Estradiol stimulates the biosynthetic pathways of breast cancer cells: Detection by metabolic flux analysis.. Metabolic Engineering.

[28] Soares R, Guo S, Gärtner F, Schmitt F, Russo J (2003). 17β-Estradiol-mediated vessel assembly and stabilization in tumor angiogenesis requires TGFβ and EGFR crosstalk.. Angiogenesis.

[29] Gourdy P, Schambourg A, Filipe C, Douin-Echinard V, Garmy-Susini B (2007). Transforming growth factor activity is a key determinant for the effect of estradiol on fatty streak deposit in hypercholesterolemic mice.. Arterioscler Thromb Vasc Biol.

[30] Djurovic S, Os I, Hofstad AE, Abdelnoor M, Westheim A (2000). Increased plasma concentrations of TGF-beta1 after hormone replacement therapy.. J Int Med.

[31] Toth B, Scholz C, Saadat G, Geller A, Schulze S (2008). Estrogen receptor modulators and estrogen receptor beta immunolabelling in human umbilical vein endothelial cells.. Acta Histochemica.

[32] Wagner AH, Schroeter MR, Hecker M (2001). 17beta-estradiol inhibition of NADPH oxidase expression in human endothelial cells.. FASEB J.

[33] Oviedo PJ, Hermenegildo C, Tarin JJ, Cano A (2007). Raloxifene increases proliferation of human endothelial cells in association with increased gene expression of cyclins A and B1.. Fertil Steril.

[34] Harris HA (2007). Estrogen Receptor-{beta}: Recent Lessons from in Vivo Studies.. Mol Endocrinol.

[35] Kolf-Clauw M, Chevy F, Wolf C, Siliart B, Citadelle D (1996). Inhibition of 7-dehydrocholesterol reductase by the teratogen AY9944: a rat model for Smith-Lemli-Opitz syndrome.. Teratology.

[36] Witsch-Baumgartner M, Loffler J, Utermann G (2001). Mutations in the human DHCR7 gene.. Hum Mutat.

[37] Monroe DG, Secreto FJ, Subramaniam M, Getz BJ, Khosla S (2005). Estrogen Receptor {alpha} and {beta} Heterodimers Exert Unique Effects on Estrogen- and Tamoxifen-Dependent Gene Expression in Human U2OS Osteosarcoma Cells.. Mol Endocrinol.

[38] Smith WL, DeWitt DL, Garavito RM (2000). Cyclooxygenases: structural, cellular, and molecular biology.. Annu Rev Biochem.

[39] Jun SS, Chen Z, Pace MC, Shaul PW (1998). Estrogen upregulates cyclooxygenase-1 gene expression in ovine fetal pulmonary artery endothelium.. J Clin Invest.

[40] Hermenegildo C, Oviedo PJ, Cano A (2006). Cyclooxygenases regulation by estradiol on endothelium.. Curr Pharm Des.

[41] Rupnow HL, Phernetton TM, Modrick ML, Wiltbank MC, Bird IM (2002). Endothelial vasodilator production by uterine and systemic arteries. VIII. Estrogen and progesterone effects on cPLA2, COX-1, and PGIS protein expression.. Biol Reprod.

[42] Farina MG, Billi S, Leguizamon G, Weissmann C, Guadagnoli T (2007). Secretory and cytosolic phospholipase A2 activities and expression are regulated by oxytocin and estradiol during labor.. Reproduction.

[43] Hermenegildo C, Garcia-Martinez MC, Tarin JJ, Cano A (2002). Estradiol reduces F2alpha -isoprostane production in cultured human endothelial cells.. Am J Physiol Heart Circ Physiol.

[44] Cicatiello L, Scafoglio C, Altucci L, Cancemi M, Natoli G (2004). A genomic view of estrogen actions in human breast cancer cells by expression profiling of the hormone-responsive transcriptome.. J Mol Endocrinol.

[45] Stevenson JC (2007). HRT and the primary prevention of cardiovascular disease.. Maturitas.

